# Supplementary material for: Genome-wide identification, characterization and gene expression of BES1 transcription factor family in grapevine (Vitis vinifera L.)
Source: Sci Rep. 2023 Jan 5;13:240. doi: 10.1038/s41598-022-24407-y (PMC9816167; doi:10.1038/s41598-022-24407-y)
Supplement: Supplementary file 3 — Supplementary Information. [file 41598_2022_24407_MOESM3_ESM.zip › Vvi_Vvi/Vitis_vinifera.PN40024.v4.dna_sm.toplevel.fa.vs.Vitis_vinifera.PN40024.v4.dna_sm.toplevel.fa.html/15.html]

|  |  |  |  |  |  |  |  |  |  |  |  |  |  |
| --- | --- | --- | --- | --- | --- | --- | --- | --- | --- | --- | --- | --- | --- |
| Duplication depth | Reference chromosome | Collinear blocks | | | | | | | | | | | |
| 0 | Vitvi15g04000\_t002 |  |  |  |  |  |  |  |  |  |  |  |  |
| 0 | Vitvi15g04001\_t001 |  |  |  |  |  |  |  |  |  |  |  |  |
| 0 | Vitvi15g04002\_t001 |  |  |  |  |  |  |  |  |  |  |  |  |
| 0 | Vitvi15g01219\_t001 |  |  |  |  |  |  |  |  |  |  |  |  |
| 0 | Vitvi15g00004\_t001 |  |  |  |  |  |  |  |  |  |  |  |  |
| 0 | Vitvi15g00005\_t001 |  |  |  |  |  |  |  |  |  |  |  |  |
| 0 | Vitvi15g04003\_t001 |  |  |  |  |  |  |  |  |  |  |  |  |
| 0 | Vitvi15g00006\_t001 |  |  |  |  |  |  |  |  |  |  |  |  |
| 0 | Vitvi15g04004\_t001 |  |  |  |  |  |  |  |  |  |  |  |  |
| 0 | Vitvi15g01220\_t001 |  |  |  |  |  |  |  |  |  |  |  |  |
| 0 | Vitvi15g00009\_t001 |  |  |  |  |  |  |  |  |  |  |  |  |
| 0 | Vitvi15g04005\_t001 |  |  |  |  |  |  |  |  |  |  |  |  |
| 0 | Vitvi15g04006\_t001 |  |  |  |  |  |  |  |  |  |  |  |  |
| 0 | Vitvi15g00010\_t001 |  |  |  |  |  |  |  |  |  |  |  |  |
| 0 | Vitvi15g00011\_t001 |  |  |  |  |  |  |  |  |  |  |  |  |
| 0 | Vitvi15g04007\_t001 |  |  |  |  |  |  |  |  |  |  |  |  |
| 0 | Vitvi15g00014\_t001 |  |  |  |  |  |  |  |  |  |  |  |  |
| 0 | Vitvi15g04008\_t001 |  |  |  |  |  |  |  |  |  |  |  |  |
| 0 | Vitvi15g04009\_t001 |  |  |  |  |  |  |  |  |  |  |  |  |
| 0 | Vitvi15g04010\_t001 |  |  |  |  |  |  |  |  |  |  |  |  |
| 0 | Vitvi15g04011\_t001 |  |  |  |  |  |  |  |  |  |  |  |  |
| 0 | Vitvi15g00016\_t001 |  |  |  |  |  |  |  |  |  |  |  |  |
| 0 | Vitvi15g00017\_t001 |  |  |  |  |  |  |  |  |  |  |  |  |
| 0 | Vitvi15g00018\_t001 |  |  |  |  |  |  |  |  |  |  |  |  |
| 0 | Vitvi15g04012\_t001 |  |  |  |  |  |  |  |  |  |  |  |  |
| 0 | Vitvi15g04013\_t001 |  |  |  |  |  |  |  |  |  |  |  |  |
| 0 | Vitvi15g00020\_t001 |  |  |  |  |  |  |  |  |  |  |  |  |
| 0 | Vitvi15g04014\_t001 |  |  |  |  |  |  |  |  |  |  |  |  |
| 0 | Vitvi15g00022\_t001 |  |  |  |  |  |  |  |  |  |  |  |  |
| 0 | Vitvi15g04015\_t001 |  |  |  |  |  |  |  |  |  |  |  |  |
| 0 | Vitvi15g04016\_t001 |  |  |  |  |  |  |  |  |  |  |  |  |
| 0 | Vitvi15g04017\_t001 |  |  |  |  |  |  |  |  |  |  |  |  |
| 0 | Vitvi15g04018\_t001 |  |  |  |  |  |  |  |  |  |  |  |  |
| 0 | Vitvi15g01224\_t001 |  |  |  |  |  |  |  |  |  |  |  |  |
| 0 | Vitvi15g00029\_t001 |  |  |  |  |  |  |  |  |  |  |  |  |
| 0 | Vitvi15g04019\_t001 |  |  |  |  |  |  |  |  |  |  |  |  |
| 0 | Vitvi15g04020\_t001 |  |  |  |  |  |  |  |  |  |  |  |  |
| 0 | Vitvi15g00033\_t001 |  |  |  |  |  |  |  |  |  |  |  |  |
| 0 | Vitvi15g00037\_t001 |  |  |  |  |  |  |  |  |  |  |  |  |
| 0 | Vitvi15g04021\_t001 |  |  |  |  |  |  |  |  |  |  |  |  |
| 0 | Vitvi15g01229\_t001 |  |  |  |  |  |  |  |  |  |  |  |  |
| 0 | Vitvi15g04022\_t001 |  |  |  |  |  |  |  |  |  |  |  |  |
| 0 | Vitvi15g00039\_t001 |  |  |  |  |  |  |  |  |  |  |  |  |
| 0 | Vitvi15g04023\_t001 |  |  |  |  |  |  |  |  |  |  |  |  |
| 0 | Vitvi15g04024\_t001 |  |  |  |  |  |  |  |  |  |  |  |  |
| 0 | Vitvi15g04025\_t001 |  |  |  |  |  |  |  |  |  |  |  |  |
| 0 | Vitvi15g04026\_t001 |  |  |  |  |  |  |  |  |  |  |  |  |
| 0 | Vitvi15g00044\_t001 |  |  |  |  |  |  |  |  |  |  |  |  |
| 0 | Vitvi15g00046\_t001 |  |  |  |  |  |  |  |  |  |  |  |  |
| 0 | Vitvi15g00047\_t001 |  |  |  |  |  |  |  |  |  |  |  |  |
| 0 | Vitvi15g00048\_t001 |  |  |  |  |  |  |  |  |  |  |  |  |
| 0 | Vitvi15g04027\_t001 |  |  |  |  |  |  |  |  |  |  |  |  |
| 0 | Vitvi15g04028\_t001 |  |  |  |  |  |  |  |  |  |  |  |  |
| 0 | Vitvi15g01233\_t001 |  |  |  |  |  |  |  |  |  |  |  |  |
| 0 | Vitvi15g04029\_t001 |  |  |  |  |  |  |  |  |  |  |  |  |
| 0 | Vitvi15g00050\_t001 |  |  |  |  |  |  |  |  |  |  |  |  |
| 0 | Vitvi15g04030\_t001 |  |  |  |  |  |  |  |  |  |  |  |  |
| 0 | Vitvi15g04031\_t001 |  |  |  |  |  |  |  |  |  |  |  |  |
| 0 | Vitvi15g00052\_t001 |  |  |  |  |  |  |  |  |  |  |  |  |
| 0 | Vitvi15g04032\_t001 |  |  |  |  |  |  |  |  |  |  |  |  |
| 0 | Vitvi15g04033\_t001 |  |  |  |  |  |  |  |  |  |  |  |  |
| 0 | Vitvi15g04034\_t001 |  |  |  |  |  |  |  |  |  |  |  |  |
| 0 | Vitvi15g04035\_t001 |  |  |  |  |  |  |  |  |  |  |  |  |
| 0 | Vitvi15g04036\_t001 |  |  |  |  |  |  |  |  |  |  |  |  |
| 0 | Vitvi15g00062\_t001 |  |  |  |  |  |  |  |  |  |  |  |  |
| 0 | Vitvi15g04037\_t001 |  |  |  |  |  |  |  |  |  |  |  |  |
| 0 | Vitvi15g04038\_t001 |  |  |  |  |  |  |  |  |  |  |  |  |
| 0 | Vitvi15g01235\_t001 |  |  |  |  |  |  |  |  |  |  |  |  |
| 0 | Vitvi15g04039\_t001 |  |  |  |  |  |  |  |  |  |  |  |  |
| 0 | Vitvi15g04040\_t001 |  |  |  |  |  |  |  |  |  |  |  |  |
| 0 | Vitvi15g00068\_t001 |  |  |  |  |  |  |  |  |  |  |  |  |
| 0 | Vitvi15g04041\_t001 |  |  |  |  |  |  |  |  |  |  |  |  |
| 0 | Vitvi15g00070\_t001 |  |  |  |  |  |  |  |  |  |  |  |  |
| 0 | Vitvi15g00071\_t001 |  |  |  |  |  |  |  |  |  |  |  |  |
| 0 | Vitvi15g04042\_t001 |  |  |  |  |  |  |  |  |  |  |  |  |
| 0 | Vitvi15g04043\_t001 |  |  |  |  |  |  |  |  |  |  |  |  |
| 0 | Vitvi15g04044\_t001 |  |  |  |  |  |  |  |  |  |  |  |  |
| 0 | Vitvi15g04045\_t001 |  |  |  |  |  |  |  |  |  |  |  |  |
| 0 | Vitvi15g00073\_t001 |  |  |  |  |  |  |  |  |  |  |  |  |
| 0 | Vitvi15g04046\_t001 |  |  |  |  |  |  |  |  |  |  |  |  |
| 0 | Vitvi15g01239\_t003 |  |  |  |  |  |  |  |  |  |  |  |  |
| 0 | Vitvi15g04047\_t001 |  |  |  |  |  |  |  |  |  |  |  |  |
| 0 | Vitvi15g00076\_t001 |  |  |  |  |  |  |  |  |  |  |  |  |
| 0 | Vitvi15g04048\_t001 |  |  |  |  |  |  |  |  |  |  |  |  |
| 0 | Vitvi15g01240\_t001 |  |  |  |  |  |  |  |  |  |  |  |  |
| 0 | Vitvi15g00079\_t001 |  |  |  |  |  |  |  |  |  |  |  |  |
| 0 | Vitvi15g00080\_t001 |  |  |  |  |  |  |  |  |  |  |  |  |
| 0 | Vitvi15g00082\_t001 |  |  |  |  |  |  |  |  |  |  |  |  |
| 0 | Vitvi15g01243\_t001 |  |  |  |  |  |  |  |  |  |  |  |  |
| 0 | Vitvi15g00084\_t001 |  |  |  |  |  |  |  |  |  |  |  |  |
| 0 | Vitvi15g01244\_t001 |  |  |  |  |  |  |  |  |  |  |  |  |
| 0 | Vitvi15g01245\_t001 |  |  |  |  |  |  |  |  |  |  |  |  |
| 0 | Vitvi15g04049\_t001 |  |  |  |  |  |  |  |  |  |  |  |  |
| 0 | Vitvi15g00089\_t001 |  |  |  |  |  |  |  |  |  |  |  |  |
| 0 | Vitvi15g04050\_t001 |  |  |  |  |  |  |  |  |  |  |  |  |
| 0 | Vitvi15g04051\_t001 |  |  |  |  |  |  |  |  |  |  |  |  |
| 0 | Vitvi15g01247\_t001 |  |  |  |  |  |  |  |  |  |  |  |  |
| 0 | Vitvi15g01248\_t001 |  |  |  |  |  |  |  |  |  |  |  |  |
| 0 | Vitvi15g04052\_t001 |  |  |  |  |  |  |  |  |  |  |  |  |
| 0 | Vitvi15g01251\_t001 |  |  |  |  |  |  |  |  |  |  |  |  |
| 0 | Vitvi15g04053\_t001 |  |  |  |  |  |  |  |  |  |  |  |  |
| 0 | Vitvi15g00097\_t001 |  |  |  |  |  |  |  |  |  |  |  |  |
| 0 | Vitvi15g04054\_t001 |  |  |  |  |  |  |  |  |  |  |  |  |
| 0 | Vitvi15g01252\_t001 |  |  |  |  |  |  |  |  |  |  |  |  |
| 0 | Vitvi15g00099\_t001 |  |  |  |  |  |  |  |  |  |  |  |  |
| 0 | Vitvi15g00100\_t001 |  |  |  |  |  |  |  |  |  |  |  |  |
| 0 | Vitvi15g00101\_t001 |  |  |  |  |  |  |  |  |  |  |  |  |
| 0 | Vitvi15g00102\_t001 |  |  |  |  |  |  |  |  |  |  |  |  |
| 0 | Vitvi15g04055\_t001 |  |  |  |  |  |  |  |  |  |  |  |  |
| 0 | Vitvi15g04056\_t001 |  |  |  |  |  |  |  |  |  |  |  |  |
| 0 | Vitvi15g04057\_t001 |  |  |  |  |  |  |  |  |  |  |  |  |
| 0 | Vitvi15g04058\_t001 |  |  |  |  |  |  |  |  |  |  |  |  |
| 0 | Vitvi15g00106\_t001 |  |  |  |  |  |  |  |  |  |  |  |  |
| 0 | Vitvi15g00107\_t001 |  |  |  |  |  |  |  |  |  |  |  |  |
| 0 | Vitvi15g04059\_t001 |  |  |  |  |  |  |  |  |  |  |  |  |
| 0 | Vitvi15g01256\_t001 |  |  |  |  |  |  |  |  |  |  |  |  |
| 0 | Vitvi15g04060\_t001 |  |  |  |  |  |  |  |  |  |  |  |  |
| 0 | Vitvi15g00110\_t001 |  |  |  |  |  |  |  |  |  |  |  |  |
| 0 | Vitvi15g04061\_t001 |  |  |  |  |  |  |  |  |  |  |  |  |
| 0 | Vitvi15g04062\_t001 |  |  |  |  |  |  |  |  |  |  |  |  |
| 0 | Vitvi15g04063\_t001 |  |  |  |  |  |  |  |  |  |  |  |  |
| 0 | Vitvi15g04064\_t001 |  |  |  |  |  |  |  |  |  |  |  |  |
| 0 | Vitvi15g04065\_t001 |  |  |  |  |  |  |  |  |  |  |  |  |
| 0 | Vitvi15g00116\_t001 |  |  |  |  |  |  |  |  |  |  |  |  |
| 0 | Vitvi15g04066\_t001 |  |  |  |  |  |  |  |  |  |  |  |  |
| 0 | Vitvi15g00118\_t001 |  |  |  |  |  |  |  |  |  |  |  |  |
| 0 | Vitvi15g04067\_t001 |  |  |  |  |  |  |  |  |  |  |  |  |
| 0 | Vitvi15g04068\_t001 |  |  |  |  |  |  |  |  |  |  |  |  |
| 0 | Vitvi15g01262\_t001 |  |  |  |  |  |  |  |  |  |  |  |  |
| 0 | Vitvi15g04069\_t001 |  |  |  |  |  |  |  |  |  |  |  |  |
| 0 | Vitvi15g01263\_t001 |  |  |  |  |  |  |  |  |  |  |  |  |
| 0 | Vitvi15g04070\_t001 |  |  |  |  |  |  |  |  |  |  |  |  |
| 0 | Vitvi15g00121\_t001 |  |  |  |  |  |  |  |  |  |  |  |  |
| 0 | Vitvi15g04071\_t001 |  |  |  |  |  |  |  |  |  |  |  |  |
| 0 | Vitvi15g00122\_t001 |  |  |  |  |  |  |  |  |  |  |  |  |
| 0 | Vitvi15g01264\_t001 |  |  |  |  |  |  |  |  |  |  |  |  |
| 0 | Vitvi15g04072\_t001 |  |  |  |  |  |  |  |  |  |  |  |  |
| 0 | Vitvi15g04073\_t001 |  |  |  |  |  |  |  |  |  |  |  |  |
| 0 | Vitvi15g00126\_t001 |  |  |  |  |  |  |  |  |  |  |  |  |
| 0 | Vitvi15g04074\_t001 |  |  |  |  |  |  |  |  |  |  |  |  |
| 0 | Vitvi15g04075\_t001 |  |  |  |  |  |  |  |  |  |  |  |  |
| 0 | Vitvi15g00132\_t001 |  |  |  |  |  |  |  |  |  |  |  |  |
| 0 | Vitvi15g04076\_t001 |  |  |  |  |  |  |  |  |  |  |  |  |
| 0 | Vitvi15g04077\_t001 |  |  |  |  |  |  |  |  |  |  |  |  |
| 0 | Vitvi15g00134\_t001 |  |  |  |  |  |  |  |  |  |  |  |  |
| 0 | Vitvi15g04078\_t001 |  |  |  |  |  |  |  |  |  |  |  |  |
| 0 | Vitvi15g04079\_t001 |  |  |  |  |  |  |  |  |  |  |  |  |
| 0 | Vitvi15g04080\_t001 |  |  |  |  |  |  |  |  |  |  |  |  |
| 0 | Vitvi15g04081\_t001 |  |  |  |  |  |  |  |  |  |  |  |  |
| 0 | Vitvi15g04082\_t001 |  |  |  |  |  |  |  |  |  |  |  |  |
| 0 | Vitvi15g01270\_t003 |  |  |  |  |  |  |  |  |  |  |  |  |
| 0 | Vitvi15g04083\_t001 |  |  |  |  |  |  |  |  |  |  |  |  |
| 0 | Vitvi15g01275\_t001 |  |  |  |  |  |  |  |  |  |  |  |  |
| 0 | Vitvi15g01276\_t001 |  |  |  |  |  |  |  |  |  |  |  |  |
| 0 | Vitvi15g04084\_t001 |  |  |  |  |  |  |  |  |  |  |  |  |
| 0 | Vitvi15g04085\_t001 |  |  |  |  |  |  |  |  |  |  |  |  |
| 0 | Vitvi15g04086\_t001 |  |  |  |  |  |  |  |  |  |  |  |  |
| 0 | Vitvi15g00144\_t001 |  |  |  |  |  |  |  |  |  |  |  |  |
| 0 | Vitvi15g04087\_t001 |  |  |  |  |  |  |  |  |  |  |  |  |
| 0 | Vitvi15g04088\_t001 |  |  |  |  |  |  |  |  |  |  |  |  |
| 0 | Vitvi15g00146\_t001 |  |  |  |  |  |  |  |  |  |  |  |  |
| 0 | Vitvi15g00148\_t001 |  |  |  |  |  |  |  |  |  |  |  |  |
| 0 | Vitvi15g00149\_t002 |  |  |  |  |  |  |  |  |  |  |  |  |
| 0 | Vitvi15g04089\_t001 |  |  |  |  |  |  |  |  |  |  |  |  |
| 0 | Vitvi15g04090\_t001 |  |  |  |  |  |  |  |  |  |  |  |  |
| 0 | Vitvi15g04091\_t001 |  |  |  |  |  |  |  |  |  |  |  |  |
| 0 | Vitvi15g04092\_t001 |  |  |  |  |  |  |  |  |  |  |  |  |
| 0 | Vitvi15g04093\_t001 |  |  |  |  |  |  |  |  |  |  |  |  |
| 0 | Vitvi15g00152\_t001 |  |  |  |  |  |  |  |  |  |  |  |  |
| 0 | Vitvi15g00153\_t001 |  |  |  |  |  |  |  |  |  |  |  |  |
| 0 | Vitvi15g04094\_t001 |  |  |  |  |  |  |  |  |  |  |  |  |
| 0 | Vitvi15g00155\_t001 |  |  |  |  |  |  |  |  |  |  |  |  |
| 0 | Vitvi15g00156\_t001 |  |  |  |  |  |  |  |  |  |  |  |  |
| 0 | Vitvi15g01277\_t001 |  |  |  |  |  |  |  |  |  |  |  |  |
| 0 | Vitvi15g04095\_t001 |  |  |  |  |  |  |  |  |  |  |  |  |
| 0 | Vitvi15g04096\_t001 |  |  |  |  |  |  |  |  |  |  |  |  |
| 0 | Vitvi15g04097\_t001 |  |  |  |  |  |  |  |  |  |  |  |  |
| 0 | Vitvi15g04098\_t001 |  |  |  |  |  |  |  |  |  |  |  |  |
| 0 | Vitvi15g04099\_t001 |  |  |  |  |  |  |  |  |  |  |  |  |
| 0 | Vitvi15g00160\_t001 |  |  |  |  |  |  |  |  |  |  |  |  |
| 0 | Vitvi15g04100\_t001 |  |  |  |  |  |  |  |  |  |  |  |  |
| 0 | Vitvi15g04101\_t001 |  |  |  |  |  |  |  |  |  |  |  |  |
| 0 | Vitvi15g04102\_t001 |  |  |  |  |  |  |  |  |  |  |  |  |
| 0 | Vitvi15g00162\_t001 |  |  |  |  |  |  |  |  |  |  |  |  |
| 0 | Vitvi15g04103\_t001 |  |  |  |  |  |  |  |  |  |  |  |  |
| 0 | Vitvi15g04104\_t001 |  |  |  |  |  |  |  |  |  |  |  |  |
| 0 | Vitvi15g04105\_t001 |  |  |  |  |  |  |  |  |  |  |  |  |
| 0 | Vitvi15g04106\_t001 |  |  |  |  |  |  |  |  |  |  |  |  |
| 0 | Vitvi15g04107\_t001 |  |  |  |  |  |  |  |  |  |  |  |  |
| 0 | Vitvi15g04108\_t001 |  |  |  |  |  |  |  |  |  |  |  |  |
| 0 | Vitvi15g04109\_t001 |  |  |  |  |  |  |  |  |  |  |  |  |
| 0 | Vitvi15g04110\_t001 |  |  |  |  |  |  |  |  |  |  |  |  |
| 0 | Vitvi15g04111\_t001 |  |  |  |  |  |  |  |  |  |  |  |  |
| 0 | Vitvi15g04112\_t001 |  |  |  |  |  |  |  |  |  |  |  |  |
| 0 | Vitvi15g04113\_t001 |  |  |  |  |  |  |  |  |  |  |  |  |
| 0 | Vitvi15g04114\_t001 |  |  |  |  |  |  |  |  |  |  |  |  |
| 0 | Vitvi15g04115\_t001 |  |  |  |  |  |  |  |  |  |  |  |  |
| 0 | Vitvi15g04116\_t001 |  |  |  |  |  |  |  |  |  |  |  |  |
| 0 | Vitvi15g00169\_t001 |  |  |  |  |  |  |  |  |  |  |  |  |
| 0 | Vitvi15g04117\_t001 |  |  |  |  |  |  |  |  |  |  |  |  |
| 0 | Vitvi15g04118\_t001 |  |  |  |  |  |  |  |  |  |  |  |  |
| 0 | Vitvi15g04119\_t001 |  |  |  |  |  |  |  |  |  |  |  |  |
| 0 | Vitvi15g04120\_t001 |  |  |  |  |  |  |  |  |  |  |  |  |
| 0 | Vitvi15g04121\_t001 |  |  |  |  |  |  |  |  |  |  |  |  |
| 0 | Vitvi15g04122\_t001 |  |  |  |  |  |  |  |  |  |  |  |  |
| 0 | Vitvi15g00172\_t001 |  |  |  |  |  |  |  |  |  |  |  |  |
| 0 | Vitvi15g04123\_t001 |  |  |  |  |  |  |  |  |  |  |  |  |
| 0 | Vitvi15g00178\_t001 |  |  |  |  |  |  |  |  |  |  |  |  |
| 0 | Vitvi15g04124\_t001 |  |  |  |  |  |  |  |  |  |  |  |  |
| 0 | Vitvi15g04125\_t001 |  |  |  |  |  |  |  |  |  |  |  |  |
| 0 | Vitvi15g00183\_t001 |  |  |  |  |  |  |  |  |  |  |  |  |
| 0 | Vitvi15g00184\_t001 |  |  |  |  |  |  |  |  |  |  |  |  |
| 0 | Vitvi15g00185\_t001 |  |  |  |  |  |  |  |  |  |  |  |  |
| 0 | Vitvi15g04126\_t001 |  |  |  |  |  |  |  |  |  |  |  |  |
| 0 | Vitvi15g01287\_t001 |  |  |  |  |  |  |  |  |  |  |  |  |
| 0 | Vitvi15g00188\_t001 |  |  |  |  |  |  |  |  |  |  |  |  |
| 0 | Vitvi15g04127\_t001 |  |  |  |  |  |  |  |  |  |  |  |  |
| 0 | Vitvi15g04128\_t001 |  |  |  |  |  |  |  |  |  |  |  |  |
| 0 | Vitvi15g00190\_t001 |  |  |  |  |  |  |  |  |  |  |  |  |
| 0 | Vitvi15g04129\_t001 |  |  |  |  |  |  |  |  |  |  |  |  |
| 0 | Vitvi15g01291\_t001 |  |  |  |  |  |  |  |  |  |  |  |  |
| 0 | Vitvi15g00194\_t001 |  |  |  |  |  |  |  |  |  |  |  |  |
| 0 | Vitvi15g00196\_t001 |  |  |  |  |  |  |  |  |  |  |  |  |
| 0 | Vitvi15g01294\_t001 |  |  |  |  |  |  |  |  |  |  |  |  |
| 0 | Vitvi15g04130\_t001 |  |  |  |  |  |  |  |  |  |  |  |  |
| 0 | Vitvi15g04131\_t001 |  |  |  |  |  |  |  |  |  |  |  |  |
| 0 | Vitvi15g04132\_t001 |  |  |  |  |  |  |  |  |  |  |  |  |
| 0 | Vitvi15g04133\_t001 |  |  |  |  |  |  |  |  |  |  |  |  |
| 0 | Vitvi15g04134\_t001 |  |  |  |  |  |  |  |  |  |  |  |  |
| 0 | Vitvi15g04135\_t001 |  |  |  |  |  |  |  |  |  |  |  |  |
| 0 | Vitvi15g04136\_t001 |  |  |  |  |  |  |  |  |  |  |  |  |
| 0 | Vitvi15g04137\_t001 |  |  |  |  |  |  |  |  |  |  |  |  |
| 0 | Vitvi15g04138\_t001 |  |  |  |  |  |  |  |  |  |  |  |  |
| 0 | Vitvi15g04139\_t001 |  |  |  |  |  |  |  |  |  |  |  |  |
| 0 | Vitvi15g00209\_t001 |  |  |  |  |  |  |  |  |  |  |  |  |
| 0 | Vitvi15g00210\_t001 |  |  |  |  |  |  |  |  |  |  |  |  |
| 0 | Vitvi15g04140\_t001 |  |  |  |  |  |  |  |  |  |  |  |  |
| 0 | Vitvi15g04141\_t001 |  |  |  |  |  |  |  |  |  |  |  |  |
| 0 | Vitvi15g04142\_t001 |  |  |  |  |  |  |  |  |  |  |  |  |
| 0 | Vitvi15g04143\_t001 |  |  |  |  |  |  |  |  |  |  |  |  |
| 0 | Vitvi15g00217\_t001 |  |  |  |  |  |  |  |  |  |  |  |  |
| 0 | Vitvi15g04144\_t001 |  |  |  |  |  |  |  |  |  |  |  |  |
| 0 | Vitvi15g00219\_t001 |  |  |  |  |  |  |  |  |  |  |  |  |
| 0 | Vitvi15g04145\_t001 |  |  |  |  |  |  |  |  |  |  |  |  |
| 0 | Vitvi15g04146\_t001 |  |  |  |  |  |  |  |  |  |  |  |  |
| 0 | Vitvi15g01213\_t001 |  |  |  |  |  |  |  |  |  |  |  |  |
| 0 | Vitvi15g01767\_t001 |  |  |  |  |  |  |  |  |  |  |  |  |
| 0 | Vitvi15g04147\_t001 |  |  |  |  |  |  |  |  |  |  |  |  |
| 0 | Vitvi15g04148\_t001 |  |  |  |  |  |  |  |  |  |  |  |  |
| 0 | Vitvi15g04149\_t001 |  |  |  |  |  |  |  |  |  |  |  |  |
| 0 | Vitvi15g04150\_t001 |  |  |  |  |  |  |  |  |  |  |  |  |
| 0 | Vitvi15g04151\_t001 |  |  |  |  |  |  |  |  |  |  |  |  |
| 0 | Vitvi15g04152\_t001 |  |  |  |  |  |  |  |  |  |  |  |  |
| 0 | Vitvi15g04153\_t001 |  |  |  |  |  |  |  |  |  |  |  |  |
| 0 | Vitvi15g04154\_t001 |  |  |  |  |  |  |  |  |  |  |  |  |
| 0 | Vitvi15g04155\_t001 |  |  |  |  |  |  |  |  |  |  |  |  |
| 0 | Vitvi15g04156\_t001 |  |  |  |  |  |  |  |  |  |  |  |  |
| 0 | Vitvi15g04157\_t001 |  |  |  |  |  |  |  |  |  |  |  |  |
| 0 | Vitvi15g04158\_t001 |  |  |  |  |  |  |  |  |  |  |  |  |
| 0 | Vitvi15g04159\_t001 |  |  |  |  |  |  |  |  |  |  |  |  |
| 0 | Vitvi15g04160\_t001 |  |  |  |  |  |  |  |  |  |  |  |  |
| 0 | Vitvi15g04161\_t001 |  |  |  |  |  |  |  |  |  |  |  |  |
| 0 | Vitvi15g04162\_t001 |  |  |  |  |  |  |  |  |  |  |  |  |
| 0 | Vitvi15g04163\_t001 |  |  |  |  |  |  |  |  |  |  |  |  |
| 0 | Vitvi15g04164\_t001 |  |  |  |  |  |  |  |  |  |  |  |  |
| 0 | Vitvi15g04165\_t001 |  |  |  |  |  |  |  |  |  |  |  |  |
| 0 | Vitvi15g04166\_t001 |  |  |  |  |  |  |  |  |  |  |  |  |
| 0 | Vitvi15g04167\_t001 |  |  |  |  |  |  |  |  |  |  |  |  |
| 0 | Vitvi15g04168\_t001 |  |  |  |  |  |  |  |  |  |  |  |  |
| 0 | Vitvi15g04169\_t001 |  |  |  |  |  |  |  |  |  |  |  |  |
| 0 | Vitvi15g04170\_t001 |  |  |  |  |  |  |  |  |  |  |  |  |
| 0 | Vitvi15g04171\_t001 |  |  |  |  |  |  |  |  |  |  |  |  |
| 0 | Vitvi15g04172\_t001 |  |  |  |  |  |  |  |  |  |  |  |  |
| 0 | Vitvi15g04173\_t001 |  |  |  |  |  |  |  |  |  |  |  |  |
| 0 | Vitvi15g04174\_t001 |  |  |  |  |  |  |  |  |  |  |  |  |
| 0 | Vitvi15g04175\_t001 |  |  |  |  |  |  |  |  |  |  |  |  |
| 0 | Vitvi15g04176\_t001 |  |  |  |  |  |  |  |  |  |  |  |  |
| 0 | Vitvi15g04177\_t001 |  |  |  |  |  |  |  |  |  |  |  |  |
| 0 | Vitvi15g01747\_t001 |  |  |  |  |  |  |  |  |  |  |  |  |
| 0 | Vitvi15g01722\_t001 |  |  |  |  |  |  |  |  |  |  |  |  |
| 0 | Vitvi15g04178\_t001 |  |  |  |  |  |  |  |  |  |  |  |  |
| 0 | Vitvi15g04179\_t001 |  |  |  |  |  |  |  |  |  |  |  |  |
| 0 | Vitvi15g04180\_t001 |  |  |  |  |  |  |  |  |  |  |  |  |
| 0 | Vitvi15g01728\_t001 |  |  |  |  |  |  |  |  |  |  |  |  |
| 0 | Vitvi15g01730\_t001 |  |  |  |  |  |  |  |  |  |  |  |  |
| 0 | Vitvi15g04181\_t001 |  |  |  |  |  |  |  |  |  |  |  |  |
| 0 | Vitvi15g01733\_t001 |  |  |  |  |  |  |  |  |  |  |  |  |
| 0 | Vitvi15g01736\_t001 |  |  |  |  |  |  |  |  |  |  |  |  |
| 0 | Vitvi15g01737\_t001 |  |  |  |  |  |  |  |  |  |  |  |  |
| 0 | Vitvi15g04183\_t001 |  |  |  |  |  |  |  |  |  |  |  |  |
| 0 | Vitvi15g04184\_t001 |  |  |  |  |  |  |  |  |  |  |  |  |
| 0 | Vitvi15g04185\_t001 |  |  |  |  |  |  |  |  |  |  |  |  |
| 0 | Vitvi15g04186\_t001 |  |  |  |  |  |  |  |  |  |  |  |  |
| 0 | Vitvi15g01753\_t001 |  |  |  |  |  |  |  |  |  |  |  |  |
| 0 | Vitvi15g04187\_t001 |  |  |  |  |  |  |  |  |  |  |  |  |
| 0 | Vitvi15g01752\_t001 |  |  |  |  |  |  |  |  |  |  |  |  |
| 0 | Vitvi15g04188\_t001 |  |  |  |  |  |  |  |  |  |  |  |  |
| 0 | Vitvi15g04189\_t001 |  |  |  |  |  |  |  |  |  |  |  |  |
| 0 | Vitvi15g01749\_t001 |  |  |  |  |  |  |  |  |  |  |  |  |
| 0 | Vitvi15g01772\_t001 |  |  |  |  |  |  |  |  |  |  |  |  |
| 0 | Vitvi15g04190\_t001 |  |  |  |  |  |  |  |  |  |  |  |  |
| 0 | Vitvi15g04191\_t001 |  |  |  |  |  |  |  |  |  |  |  |  |
| 0 | Vitvi15g04192\_t001 |  |  |  |  |  |  |  |  |  |  |  |  |
| 0 | Vitvi15g01303\_t001 |  |  |  |  |  |  |  |  |  |  |  |  |
| 0 | Vitvi15g01305\_t001 |  |  |  |  |  |  |  |  |  |  |  |  |
| 0 | Vitvi15g00224\_t001 |  |  |  |  |  |  |  |  |  |  |  |  |
| 0 | Vitvi15g04193\_t001 |  |  |  |  |  |  |  |  |  |  |  |  |
| 0 | Vitvi15g01306\_t001 |  |  |  |  |  |  |  |  |  |  |  |  |
| 0 | Vitvi15g04194\_t001 |  |  |  |  |  |  |  |  |  |  |  |  |
| 0 | Vitvi15g04195\_t001 |  |  |  |  |  |  |  |  |  |  |  |  |
| 0 | Vitvi15g00225\_t002 |  |  |  |  |  |  |  |  |  |  |  |  |
| 0 | Vitvi15g04196\_t001 |  |  |  |  |  |  |  |  |  |  |  |  |
| 0 | Vitvi15g04197\_t001 |  |  |  |  |  |  |  |  |  |  |  |  |
| 0 | Vitvi15g00231\_t001 |  |  |  |  |  |  |  |  |  |  |  |  |
| 0 | Vitvi15g04198\_t001 |  |  |  |  |  |  |  |  |  |  |  |  |
| 0 | Vitvi15g01308\_t001 |  |  |  |  |  |  |  |  |  |  |  |  |
| 0 | Vitvi15g01309\_t001 |  |  |  |  |  |  |  |  |  |  |  |  |
| 0 | Vitvi15g04199\_t001 |  |  |  |  |  |  |  |  |  |  |  |  |
| 0 | Vitvi15g04200\_t001 |  |  |  |  |  |  |  |  |  |  |  |  |
| 0 | Vitvi15g00237\_t001 |  |  |  |  |  |  |  |  |  |  |  |  |
| 0 | Vitvi15g01313\_t001 |  |  |  |  |  |  |  |  |  |  |  |  |
| 0 | Vitvi15g00239\_t001 |  |  |  |  |  |  |  |  |  |  |  |  |
| 0 | Vitvi15g00240\_t001 |  |  |  |  |  |  |  |  |  |  |  |  |
| 0 | Vitvi15g00241\_t001 |  |  |  |  |  |  |  |  |  |  |  |  |
| 0 | Vitvi15g00244\_t001 |  |  |  |  |  |  |  |  |  |  |  |  |
| 0 | Vitvi15g04201\_t001 |  |  |  |  |  |  |  |  |  |  |  |  |
| 0 | Vitvi15g00247\_t001 |  |  |  |  |  |  |  |  |  |  |  |  |
| 0 | Vitvi15g00248\_t001 |  |  |  |  |  |  |  |  |  |  |  |  |
| 0 | Vitvi15g00250\_t001 |  |  |  |  |  |  |  |  |  |  |  |  |
| 0 | Vitvi15g00251\_t001 |  |  |  |  |  |  |  |  |  |  |  |  |
| 0 | Vitvi15g04202\_t001 |  |  |  |  |  |  |  |  |  |  |  |  |
| 0 | Vitvi15g04203\_t001 |  |  |  |  |  |  |  |  |  |  |  |  |
| 0 | Vitvi15g04204\_t001 |  |  |  |  |  |  |  |  |  |  |  |  |
| 0 | Vitvi15g04205\_t001 |  |  |  |  |  |  |  |  |  |  |  |  |
| 0 | Vitvi15g01317\_t001 |  |  |  |  |  |  |  |  |  |  |  |  |
| 0 | Vitvi15g00254\_t001 |  |  |  |  |  |  |  |  |  |  |  |  |
| 0 | Vitvi15g00257\_t001 |  |  |  |  |  |  |  |  |  |  |  |  |
| 0 | Vitvi15g04206\_t001 |  |  |  |  |  |  |  |  |  |  |  |  |
| 0 | Vitvi15g00258\_t001 |  |  |  |  |  |  |  |  |  |  |  |  |
| 0 | Vitvi15g00260\_t001 |  |  |  |  |  |  |  |  |  |  |  |  |
| 0 | Vitvi15g01318\_t001 |  |  |  |  |  |  |  |  |  |  |  |  |
| 0 | Vitvi15g04207\_t001 |  |  |  |  |  |  |  |  |  |  |  |  |
| 0 | Vitvi15g04208\_t001 |  |  |  |  |  |  |  |  |  |  |  |  |
| 0 | Vitvi15g00263\_t001 |  |  |  |  |  |  |  |  |  |  |  |  |
| 0 | Vitvi15g04209\_t001 |  |  |  |  |  |  |  |  |  |  |  |  |
| 1 | Vitvi15g04210\_t001 |  | Vitvi16g04189\_t001 |  |  |  |  |  |  |  |  |  |  |
| 1 | Vitvi15g04211\_t001 |  | Vitvi16g04190\_t001 |  |  |  |  |  |  |  |  |  |  |
| 1 | Vitvi15g04212\_t001 |  | Vitvi16g04191\_t001 |  |  |  |  |  |  |  |  |  |  |
| 1 | Vitvi15g04213\_t001 |  | \| \| |  |  |  |  |  |  |  |  |  |  |
| 1 | Vitvi15g04214\_t001 |  | \| \| |  |  |  |  |  |  |  |  |  |  |
| 1 | Vitvi15g04215\_t001 |  | Vitvi16g04193\_t001 |  |  |  |  |  |  |  |  |  |  |
| 1 | Vitvi15g04216\_t001 |  | \| \| |  |  |  |  |  |  |  |  |  |  |
| 1 | Vitvi15g04217\_t001 |  | Vitvi16g04198\_t001 |  |  |  |  |  |  |  |  |  |  |
| 1 | Vitvi15g04218\_t001 |  | \| \| |  |  |  |  |  |  |  |  |  |  |
| 1 | Vitvi15g01325\_t001 |  | \| \| |  |  |  |  |  |  |  |  |  |  |
| 1 | Vitvi15g00267\_t001 |  | \| \| |  |  |  |  |  |  |  |  |  |  |
| 1 | Vitvi15g04219\_t001 |  | \| \| |  |  |  |  |  |  |  |  |  |  |
| 1 | Vitvi15g04220\_t001 |  | \| \| |  |  |  |  |  |  |  |  |  |  |
| 1 | Vitvi15g04221\_t001 |  | \| \| |  |  |  |  |  |  |  |  |  |  |
| 1 | Vitvi15g00269\_t001 |  | \| \| |  |  |  |  |  |  |  |  |  |  |
| 1 | Vitvi15g01327\_t001 |  | \| \| |  |  |  |  |  |  |  |  |  |  |
| 1 | Vitvi15g00271\_t001 |  | \| \| |  |  |  |  |  |  |  |  |  |  |
| 1 | Vitvi15g04222\_t001 |  | \| \| |  |  |  |  |  |  |  |  |  |  |
| 1 | Vitvi15g04223\_t001 |  | \| \| |  |  |  |  |  |  |  |  |  |  |
| 1 | Vitvi15g04224\_t001 |  | \| \| |  |  |  |  |  |  |  |  |  |  |
| 1 | Vitvi15g00275\_t001 |  | Vitvi16g04208\_t001 |  |  |  |  |  |  |  |  |  |  |
| 0 | Vitvi15g04225\_t001 |  |  |  |  |  |  |  |  |  |  |  |  |
| 0 | Vitvi15g00279\_t001 |  |  |  |  |  |  |  |  |  |  |  |  |
| 0 | Vitvi15g04226\_t001 |  |  |  |  |  |  |  |  |  |  |  |  |
| 0 | Vitvi15g04227\_t001 |  |  |  |  |  |  |  |  |  |  |  |  |
| 0 | Vitvi15g04228\_t001 |  |  |  |  |  |  |  |  |  |  |  |  |
| 0 | Vitvi15g00282\_t001 |  |  |  |  |  |  |  |  |  |  |  |  |
| 0 | Vitvi15g04229\_t001 |  |  |  |  |  |  |  |  |  |  |  |  |
| 0 | Vitvi15g04230\_t001 |  |  |  |  |  |  |  |  |  |  |  |  |
| 0 | Vitvi15g00285\_t001 |  |  |  |  |  |  |  |  |  |  |  |  |
| 0 | Vitvi15g00287\_t001 |  |  |  |  |  |  |  |  |  |  |  |  |
| 0 | Vitvi15g00289\_t003 |  |  |  |  |  |  |  |  |  |  |  |  |
| 0 | Vitvi15g00291\_t001 |  |  |  |  |  |  |  |  |  |  |  |  |
| 0 | Vitvi15g04231\_t001 |  |  |  |  |  |  |  |  |  |  |  |  |
| 0 | Vitvi15g00292\_t001 |  |  |  |  |  |  |  |  |  |  |  |  |
| 0 | Vitvi15g04232\_t001 |  |  |  |  |  |  |  |  |  |  |  |  |
| 0 | Vitvi15g04233\_t001 |  |  |  |  |  |  |  |  |  |  |  |  |
| 0 | Vitvi15g01344\_t001 |  |  |  |  |  |  |  |  |  |  |  |  |
| 0 | Vitvi15g00296\_t001 |  |  |  |  |  |  |  |  |  |  |  |  |
| 0 | Vitvi15g04234\_t001 |  |  |  |  |  |  |  |  |  |  |  |  |
| 0 | Vitvi15g00298\_t001 |  |  |  |  |  |  |  |  |  |  |  |  |
| 0 | Vitvi15g04235\_t001 |  |  |  |  |  |  |  |  |  |  |  |  |
| 0 | Vitvi15g04236\_t001 |  |  |  |  |  |  |  |  |  |  |  |  |
| 0 | Vitvi15g00300\_t001 |  |  |  |  |  |  |  |  |  |  |  |  |
| 0 | Vitvi15g04237\_t001 |  |  |  |  |  |  |  |  |  |  |  |  |
| 0 | Vitvi15g00301\_t001 |  |  |  |  |  |  |  |  |  |  |  |  |
| 0 | Vitvi15g00304\_t001 |  |  |  |  |  |  |  |  |  |  |  |  |
| 0 | Vitvi15g00306\_t001 |  |  |  |  |  |  |  |  |  |  |  |  |
| 0 | Vitvi15g00307\_t001 |  |  |  |  |  |  |  |  |  |  |  |  |
| 0 | Vitvi15g00308\_t001 |  |  |  |  |  |  |  |  |  |  |  |  |
| 0 | Vitvi15g00310\_t001 |  |  |  |  |  |  |  |  |  |  |  |  |
| 0 | Vitvi15g00311\_t001 |  |  |  |  |  |  |  |  |  |  |  |  |
| 0 | Vitvi15g04238\_t001 |  |  |  |  |  |  |  |  |  |  |  |  |
| 0 | Vitvi15g01350\_t001 |  |  |  |  |  |  |  |  |  |  |  |  |
| 0 | Vitvi15g01351\_t001 |  |  |  |  |  |  |  |  |  |  |  |  |
| 0 | Vitvi15g04239\_t001 |  |  |  |  |  |  |  |  |  |  |  |  |
| 0 | Vitvi15g04240\_t001 |  |  |  |  |  |  |  |  |  |  |  |  |
| 0 | Vitvi15g00314\_t001 |  |  |  |  |  |  |  |  |  |  |  |  |
| 0 | Vitvi15g00317\_t001 |  |  |  |  |  |  |  |  |  |  |  |  |
| 0 | Vitvi15g04241\_t001 |  |  |  |  |  |  |  |  |  |  |  |  |
| 0 | Vitvi15g01355\_t001 |  |  |  |  |  |  |  |  |  |  |  |  |
| 0 | Vitvi15g04242\_t001 |  |  |  |  |  |  |  |  |  |  |  |  |
| 0 | Vitvi15g04243\_t001 |  |  |  |  |  |  |  |  |  |  |  |  |
| 0 | Vitvi15g00321\_t002 |  |  |  |  |  |  |  |  |  |  |  |  |
| 0 | Vitvi15g04244\_t001 |  |  |  |  |  |  |  |  |  |  |  |  |
| 0 | Vitvi15g04245\_t001 |  |  |  |  |  |  |  |  |  |  |  |  |
| 0 | Vitvi15g04246\_t001 |  |  |  |  |  |  |  |  |  |  |  |  |
| 0 | Vitvi15g04247\_t001 |  |  |  |  |  |  |  |  |  |  |  |  |
| 0 | Vitvi15g04248\_t001 |  |  |  |  |  |  |  |  |  |  |  |  |
| 0 | Vitvi15g01358\_t001 |  |  |  |  |  |  |  |  |  |  |  |  |
| 0 | Vitvi15g04249\_t001 |  |  |  |  |  |  |  |  |  |  |  |  |
| 0 | Vitvi15g04250\_t001 |  |  |  |  |  |  |  |  |  |  |  |  |
| 0 | Vitvi15g00331\_t001 |  |  |  |  |  |  |  |  |  |  |  |  |
| 0 | Vitvi15g04251\_t001 |  |  |  |  |  |  |  |  |  |  |  |  |
| 0 | Vitvi15g04252\_t001 |  |  |  |  |  |  |  |  |  |  |  |  |
| 0 | Vitvi15g04253\_t001 |  |  |  |  |  |  |  |  |  |  |  |  |
| 0 | Vitvi15g04254\_t001 |  |  |  |  |  |  |  |  |  |  |  |  |
| 0 | Vitvi15g00335\_t001 |  |  |  |  |  |  |  |  |  |  |  |  |
| 0 | Vitvi15g04255\_t001 |  |  |  |  |  |  |  |  |  |  |  |  |
| 0 | Vitvi15g04256\_t001 |  |  |  |  |  |  |  |  |  |  |  |  |
| 0 | Vitvi15g01366\_t001 |  |  |  |  |  |  |  |  |  |  |  |  |
| 0 | Vitvi15g00341\_t001 |  |  |  |  |  |  |  |  |  |  |  |  |
| 0 | Vitvi15g04257\_t001 |  |  |  |  |  |  |  |  |  |  |  |  |
| 0 | Vitvi15g00349\_t002 |  |  |  |  |  |  |  |  |  |  |  |  |
| 0 | Vitvi15g00352\_t001 |  |  |  |  |  |  |  |  |  |  |  |  |
| 0 | Vitvi15g00357\_t001 |  |  |  |  |  |  |  |  |  |  |  |  |
| 0 | Vitvi15g01371\_t001 |  |  |  |  |  |  |  |  |  |  |  |  |
| 0 | Vitvi15g01372\_t001 |  |  |  |  |  |  |  |  |  |  |  |  |
| 0 | Vitvi15g00359\_t001 |  |  |  |  |  |  |  |  |  |  |  |  |
| 0 | Vitvi15g00360\_t001 |  |  |  |  |  |  |  |  |  |  |  |  |
| 0 | Vitvi15g00362\_t001 |  |  |  |  |  |  |  |  |  |  |  |  |
| 0 | Vitvi15g00364\_t001 |  |  |  |  |  |  |  |  |  |  |  |  |
| 0 | Vitvi15g00365\_t001 |  |  |  |  |  |  |  |  |  |  |  |  |
| 0 | Vitvi15g04258\_t001 |  |  |  |  |  |  |  |  |  |  |  |  |
| 0 | Vitvi15g00369\_t001 |  |  |  |  |  |  |  |  |  |  |  |  |
| 0 | Vitvi15g00372\_t001 |  |  |  |  |  |  |  |  |  |  |  |  |
| 0 | Vitvi15g00373\_t003 |  |  |  |  |  |  |  |  |  |  |  |  |
| 0 | Vitvi15g04259\_t001 |  |  |  |  |  |  |  |  |  |  |  |  |
| 0 | Vitvi15g00377\_t001 |  |  |  |  |  |  |  |  |  |  |  |  |
| 0 | Vitvi15g04260\_t001 |  |  |  |  |  |  |  |  |  |  |  |  |
| 0 | Vitvi15g04261\_t001 |  |  |  |  |  |  |  |  |  |  |  |  |
| 0 | Vitvi15g00380\_t001 |  |  |  |  |  |  |  |  |  |  |  |  |
| 0 | Vitvi15g00383\_t001 |  |  |  |  |  |  |  |  |  |  |  |  |
| 0 | Vitvi15g00385\_t001 |  |  |  |  |  |  |  |  |  |  |  |  |
| 0 | Vitvi15g04262\_t001 |  |  |  |  |  |  |  |  |  |  |  |  |
| 0 | Vitvi15g00388\_t001 |  |  |  |  |  |  |  |  |  |  |  |  |
| 0 | Vitvi15g00390\_t001 |  |  |  |  |  |  |  |  |  |  |  |  |
| 0 | Vitvi15g00396\_t001 |  |  |  |  |  |  |  |  |  |  |  |  |
| 0 | Vitvi15g04264\_t001 |  |  |  |  |  |  |  |  |  |  |  |  |
| 0 | Vitvi15g04265\_t001 |  |  |  |  |  |  |  |  |  |  |  |  |
| 0 | Vitvi15g01384\_t001 |  |  |  |  |  |  |  |  |  |  |  |  |
| 0 | Vitvi15g04266\_t001 |  |  |  |  |  |  |  |  |  |  |  |  |
| 0 | Vitvi15g00397\_t001 |  |  |  |  |  |  |  |  |  |  |  |  |
| 0 | Vitvi15g00399\_t001 |  |  |  |  |  |  |  |  |  |  |  |  |
| 0 | Vitvi15g01385\_t001 |  |  |  |  |  |  |  |  |  |  |  |  |
| 0 | Vitvi15g00402\_t001 |  |  |  |  |  |  |  |  |  |  |  |  |
| 0 | Vitvi15g00404\_t001 |  |  |  |  |  |  |  |  |  |  |  |  |
| 0 | Vitvi15g01387\_t001 |  |  |  |  |  |  |  |  |  |  |  |  |
| 0 | Vitvi15g00406\_t001 |  |  |  |  |  |  |  |  |  |  |  |  |
| 0 | Vitvi15g04267\_t001 |  |  |  |  |  |  |  |  |  |  |  |  |
| 0 | Vitvi15g01388\_t001 |  |  |  |  |  |  |  |  |  |  |  |  |
| 0 | Vitvi15g01389\_t001 |  |  |  |  |  |  |  |  |  |  |  |  |
| 0 | Vitvi15g04268\_t001 |  |  |  |  |  |  |  |  |  |  |  |  |
| 0 | Vitvi15g04269\_t001 |  |  |  |  |  |  |  |  |  |  |  |  |
| 0 | Vitvi15g00410\_t002 |  |  |  |  |  |  |  |  |  |  |  |  |
| 0 | Vitvi15g04270\_t001 |  |  |  |  |  |  |  |  |  |  |  |  |
| 0 | Vitvi15g01394\_t001 |  |  |  |  |  |  |  |  |  |  |  |  |
| 0 | Vitvi15g04271\_t001 |  |  |  |  |  |  |  |  |  |  |  |  |
| 0 | Vitvi15g04272\_t001 |  |  |  |  |  |  |  |  |  |  |  |  |
| 0 | Vitvi15g04273\_t001 |  |  |  |  |  |  |  |  |  |  |  |  |
| 0 | Vitvi15g01395\_t001 |  |  |  |  |  |  |  |  |  |  |  |  |
| 0 | Vitvi15g01396\_t001 |  |  |  |  |  |  |  |  |  |  |  |  |
| 0 | Vitvi15g04274\_t001 |  |  |  |  |  |  |  |  |  |  |  |  |
| 0 | Vitvi15g04275\_t001 |  |  |  |  |  |  |  |  |  |  |  |  |
| 0 | Vitvi15g04276\_t001 |  |  |  |  |  |  |  |  |  |  |  |  |
| 0 | Vitvi15g04277\_t001 |  |  |  |  |  |  |  |  |  |  |  |  |
| 0 | Vitvi15g04278\_t001 |  |  |  |  |  |  |  |  |  |  |  |  |
| 0 | Vitvi15g04279\_t001 |  |  |  |  |  |  |  |  |  |  |  |  |
| 0 | Vitvi15g00414\_t001 |  |  |  |  |  |  |  |  |  |  |  |  |
| 0 | Vitvi15g04280\_t001 |  |  |  |  |  |  |  |  |  |  |  |  |
| 0 | Vitvi15g04281\_t001 |  |  |  |  |  |  |  |  |  |  |  |  |
| 0 | Vitvi15g01399\_t001 |  |  |  |  |  |  |  |  |  |  |  |  |
| 0 | Vitvi15g00419\_t001 |  |  |  |  |  |  |  |  |  |  |  |  |
| 0 | Vitvi15g04282\_t001 |  |  |  |  |  |  |  |  |  |  |  |  |
| 0 | Vitvi15g04283\_t001 |  |  |  |  |  |  |  |  |  |  |  |  |
| 0 | Vitvi15g04284\_t001 |  |  |  |  |  |  |  |  |  |  |  |  |
| 0 | Vitvi15g00424\_t001 |  |  |  |  |  |  |  |  |  |  |  |  |
| 0 | Vitvi15g04285\_t001 |  |  |  |  |  |  |  |  |  |  |  |  |
| 0 | Vitvi15g00427\_t001 |  |  |  |  |  |  |  |  |  |  |  |  |
| 0 | Vitvi15g00428\_t001 |  |  |  |  |  |  |  |  |  |  |  |  |
| 0 | Vitvi15g04286\_t001 |  |  |  |  |  |  |  |  |  |  |  |  |
| 0 | Vitvi15g00431\_t001 |  |  |  |  |  |  |  |  |  |  |  |  |
| 0 | Vitvi15g00432\_t001 |  |  |  |  |  |  |  |  |  |  |  |  |
| 0 | Vitvi15g04287\_t001 |  |  |  |  |  |  |  |  |  |  |  |  |
| 0 | Vitvi15g00434\_t001 |  |  |  |  |  |  |  |  |  |  |  |  |
| 0 | Vitvi15g01403\_t001 |  |  |  |  |  |  |  |  |  |  |  |  |
| 0 | Vitvi15g01402\_t001 |  |  |  |  |  |  |  |  |  |  |  |  |
| 0 | Vitvi15g01405\_t001 |  |  |  |  |  |  |  |  |  |  |  |  |
| 0 | Vitvi15g01406\_t001 |  |  |  |  |  |  |  |  |  |  |  |  |
| 0 | Vitvi15g00437\_t001 |  |  |  |  |  |  |  |  |  |  |  |  |
| 0 | Vitvi15g00438\_t001 |  |  |  |  |  |  |  |  |  |  |  |  |
| 0 | Vitvi15g00440\_t001 |  |  |  |  |  |  |  |  |  |  |  |  |
| 0 | Vitvi15g04288\_t001 |  |  |  |  |  |  |  |  |  |  |  |  |
| 0 | Vitvi15g00442\_t001 |  |  |  |  |  |  |  |  |  |  |  |  |
| 0 | Vitvi15g00443\_t001 |  |  |  |  |  |  |  |  |  |  |  |  |
| 0 | Vitvi15g00445\_t001 |  |  |  |  |  |  |  |  |  |  |  |  |
| 0 | Vitvi15g04289\_t001 |  |  |  |  |  |  |  |  |  |  |  |  |
| 0 | Vitvi15g04290\_t001 |  |  |  |  |  |  |  |  |  |  |  |  |
| 0 | Vitvi15g00447\_t001 |  |  |  |  |  |  |  |  |  |  |  |  |
| 0 | Vitvi15g00448\_t001 |  |  |  |  |  |  |  |  |  |  |  |  |
| 0 | Vitvi15g04291\_t001 |  |  |  |  |  |  |  |  |  |  |  |  |
| 0 | Vitvi15g04292\_t001 |  |  |  |  |  |  |  |  |  |  |  |  |
| 0 | Vitvi15g00452\_t001 |  |  |  |  |  |  |  |  |  |  |  |  |
| 0 | Vitvi15g04293\_t001 |  |  |  |  |  |  |  |  |  |  |  |  |
| 0 | Vitvi15g04294\_t001 |  |  |  |  |  |  |  |  |  |  |  |  |
| 0 | Vitvi15g00457\_t001 |  |  |  |  |  |  |  |  |  |  |  |  |
| 0 | Vitvi15g00458\_t001 |  |  |  |  |  |  |  |  |  |  |  |  |
| 0 | Vitvi15g00459\_t001 |  |  |  |  |  |  |  |  |  |  |  |  |
| 0 | Vitvi15g01209\_t001 |  |  |  |  |  |  |  |  |  |  |  |  |
| 0 | Vitvi15g04295\_t001 |  |  |  |  |  |  |  |  |  |  |  |  |
| 0 | Vitvi15g04296\_t001 |  |  |  |  |  |  |  |  |  |  |  |  |
| 0 | Vitvi15g01210\_t001 |  |  |  |  |  |  |  |  |  |  |  |  |
| 0 | Vitvi15g00462\_t001 |  |  |  |  |  |  |  |  |  |  |  |  |
| 0 | Vitvi15g04297\_t001 |  |  |  |  |  |  |  |  |  |  |  |  |
| 0 | Vitvi15g00463\_t001 |  |  |  |  |  |  |  |  |  |  |  |  |
| 0 | Vitvi15g00465\_t001 |  |  |  |  |  |  |  |  |  |  |  |  |
| 0 | Vitvi15g04298\_t001 |  |  |  |  |  |  |  |  |  |  |  |  |
| 0 | Vitvi15g04299\_t001 |  |  |  |  |  |  |  |  |  |  |  |  |
| 0 | Vitvi15g04300\_t001 |  |  |  |  |  |  |  |  |  |  |  |  |
| 0 | Vitvi15g00472\_t001 |  |  |  |  |  |  |  |  |  |  |  |  |
| 0 | Vitvi15g00473\_t001 |  |  |  |  |  |  |  |  |  |  |  |  |
| 0 | Vitvi15g00475\_t001 |  |  |  |  |  |  |  |  |  |  |  |  |
| 0 | Vitvi15g00476\_t001 |  |  |  |  |  |  |  |  |  |  |  |  |
| 0 | Vitvi15g00477\_t002 |  |  |  |  |  |  |  |  |  |  |  |  |
| 0 | Vitvi15g00478\_t001 |  |  |  |  |  |  |  |  |  |  |  |  |
| 0 | Vitvi15g04301\_t001 |  |  |  |  |  |  |  |  |  |  |  |  |
| 0 | Vitvi15g04302\_t001 |  |  |  |  |  |  |  |  |  |  |  |  |
| 0 | Vitvi15g04303\_t001 |  |  |  |  |  |  |  |  |  |  |  |  |
| 0 | Vitvi15g00486\_t001 |  |  |  |  |  |  |  |  |  |  |  |  |
| 0 | Vitvi15g00490\_t001 |  |  |  |  |  |  |  |  |  |  |  |  |
| 0 | Vitvi15g01410\_t001 |  |  |  |  |  |  |  |  |  |  |  |  |
| 0 | Vitvi15g01413\_t001 |  |  |  |  |  |  |  |  |  |  |  |  |
| 0 | Vitvi15g04304\_t001 |  |  |  |  |  |  |  |  |  |  |  |  |
| 0 | Vitvi15g01414\_t001 |  |  |  |  |  |  |  |  |  |  |  |  |
| 0 | Vitvi15g00492\_t001 |  |  |  |  |  |  |  |  |  |  |  |  |
| 0 | Vitvi15g00494\_t001 |  |  |  |  |  |  |  |  |  |  |  |  |
| 0 | Vitvi15g04305\_t001 |  |  |  |  |  |  |  |  |  |  |  |  |
| 0 | Vitvi15g04306\_t001 |  |  |  |  |  |  |  |  |  |  |  |  |
| 0 | Vitvi15g00495\_t001 |  |  |  |  |  |  |  |  |  |  |  |  |
| 0 | Vitvi15g04307\_t001 |  |  |  |  |  |  |  |  |  |  |  |  |
| 0 | Vitvi15g04308\_t001 |  |  |  |  |  |  |  |  |  |  |  |  |
| 0 | Vitvi15g00500\_t001 |  |  |  |  |  |  |  |  |  |  |  |  |
| 0 | Vitvi15g01417\_t001 |  |  |  |  |  |  |  |  |  |  |  |  |
| 0 | Vitvi15g00502\_t001 |  |  |  |  |  |  |  |  |  |  |  |  |
| 0 | Vitvi15g00504\_t001 |  |  |  |  |  |  |  |  |  |  |  |  |
| 0 | Vitvi15g00505\_t001 |  |  |  |  |  |  |  |  |  |  |  |  |
| 0 | Vitvi15g04309\_t001 |  |  |  |  |  |  |  |  |  |  |  |  |
| 0 | Vitvi15g00509\_t001 |  |  |  |  |  |  |  |  |  |  |  |  |
| 0 | Vitvi15g00510\_t001 |  |  |  |  |  |  |  |  |  |  |  |  |
| 0 | Vitvi15g00511\_t001 |  |  |  |  |  |  |  |  |  |  |  |  |
| 0 | Vitvi15g04310\_t001 |  |  |  |  |  |  |  |  |  |  |  |  |
| 0 | Vitvi15g00513\_t003 |  |  |  |  |  |  |  |  |  |  |  |  |
| 0 | Vitvi15g00514\_t001 |  |  |  |  |  |  |  |  |  |  |  |  |
| 0 | Vitvi15g00516\_t001 |  |  |  |  |  |  |  |  |  |  |  |  |
| 0 | Vitvi15g00517\_t001 |  |  |  |  |  |  |  |  |  |  |  |  |
| 0 | Vitvi15g00518\_t001 |  |  |  |  |  |  |  |  |  |  |  |  |
| 0 | Vitvi15g00520\_t001 |  |  |  |  |  |  |  |  |  |  |  |  |
| 0 | Vitvi15g04311\_t001 |  |  |  |  |  |  |  |  |  |  |  |  |
| 0 | Vitvi15g04312\_t001 |  |  |  |  |  |  |  |  |  |  |  |  |
| 0 | Vitvi15g04313\_t001 |  |  |  |  |  |  |  |  |  |  |  |  |
| 0 | Vitvi15g04314\_t001 |  |  |  |  |  |  |  |  |  |  |  |  |
| 0 | Vitvi15g00522\_t001 |  |  |  |  |  |  |  |  |  |  |  |  |
| 0 | Vitvi15g00523\_t001 |  |  |  |  |  |  |  |  |  |  |  |  |
| 0 | Vitvi15g01420\_t002 |  |  |  |  |  |  |  |  |  |  |  |  |
| 0 | Vitvi15g04315\_t001 |  |  |  |  |  |  |  |  |  |  |  |  |
| 0 | Vitvi15g00526\_t001 |  |  |  |  |  |  |  |  |  |  |  |  |
| 0 | Vitvi15g00527\_t001 |  |  |  |  |  |  |  |  |  |  |  |  |
| 0 | Vitvi15g00528\_t001 |  |  |  |  |  |  |  |  |  |  |  |  |
| 0 | Vitvi15g04316\_t001 |  |  |  |  |  |  |  |  |  |  |  |  |
| 0 | Vitvi15g01421\_t001 |  |  |  |  |  |  |  |  |  |  |  |  |
| 0 | Vitvi15g00529\_t002 |  |  |  |  |  |  |  |  |  |  |  |  |
| 0 | Vitvi15g00531\_t001 |  |  |  |  |  |  |  |  |  |  |  |  |
| 0 | Vitvi15g00532\_t001 |  |  |  |  |  |  |  |  |  |  |  |  |
| 0 | Vitvi15g00533\_t001 |  |  |  |  |  |  |  |  |  |  |  |  |
| 0 | Vitvi15g04317\_t001 |  |  |  |  |  |  |  |  |  |  |  |  |
| 0 | Vitvi15g04318\_t001 |  |  |  |  |  |  |  |  |  |  |  |  |
| 0 | Vitvi15g01422\_t001 |  |  |  |  |  |  |  |  |  |  |  |  |
| 0 | Vitvi15g00537\_t001 |  |  |  |  |  |  |  |  |  |  |  |  |
| 0 | Vitvi15g00538\_t001 |  |  |  |  |  |  |  |  |  |  |  |  |
| 0 | Vitvi15g00539\_t001 |  |  |  |  |  |  |  |  |  |  |  |  |
| 0 | Vitvi15g00540\_t001 |  |  |  |  |  |  |  |  |  |  |  |  |
| 0 | Vitvi15g00541\_t001 |  |  |  |  |  |  |  |  |  |  |  |  |
| 0 | Vitvi15g00542\_t001 |  |  |  |  |  |  |  |  |  |  |  |  |
| 0 | Vitvi15g00543\_t001 |  |  |  |  |  |  |  |  |  |  |  |  |
| 0 | Vitvi15g00544\_t001 |  |  |  |  |  |  |  |  |  |  |  |  |
| 0 | Vitvi15g00545\_t001 |  |  |  |  |  |  |  |  |  |  |  |  |
| 0 | Vitvi15g00547\_t001 |  |  |  |  |  |  |  |  |  |  |  |  |
| 0 | Vitvi15g04319\_t001 |  |  |  |  |  |  |  |  |  |  |  |  |
| 0 | Vitvi15g00548\_t001 |  |  |  |  |  |  |  |  |  |  |  |  |
| 0 | Vitvi15g00549\_t001 |  |  |  |  |  |  |  |  |  |  |  |  |
| 0 | Vitvi15g00554\_t001 |  |  |  |  |  |  |  |  |  |  |  |  |
| 0 | Vitvi15g01425\_t001 |  |  |  |  |  |  |  |  |  |  |  |  |
| 0 | Vitvi15g04320\_t001 |  |  |  |  |  |  |  |  |  |  |  |  |
| 0 | Vitvi15g00555\_t001 |  |  |  |  |  |  |  |  |  |  |  |  |
| 0 | Vitvi15g00556\_t001 |  |  |  |  |  |  |  |  |  |  |  |  |
| 0 | Vitvi15g04321\_t001 |  |  |  |  |  |  |  |  |  |  |  |  |
| 0 | Vitvi15g00557\_t001 |  |  |  |  |  |  |  |  |  |  |  |  |
| 0 | Vitvi15g04322\_t001 |  |  |  |  |  |  |  |  |  |  |  |  |
| 0 | Vitvi15g04323\_t001 |  |  |  |  |  |  |  |  |  |  |  |  |
| 0 | Vitvi15g00562\_t001 |  |  |  |  |  |  |  |  |  |  |  |  |
| 0 | Vitvi15g00563\_t001 |  |  |  |  |  |  |  |  |  |  |  |  |
| 0 | Vitvi15g00564\_t001 |  |  |  |  |  |  |  |  |  |  |  |  |
| 0 | Vitvi15g00565\_t001 |  |  |  |  |  |  |  |  |  |  |  |  |
| 0 | Vitvi15g01202\_t001 |  |  |  |  |  |  |  |  |  |  |  |  |
| 0 | Vitvi15g01206\_t001 |  |  |  |  |  |  |  |  |  |  |  |  |
| 0 | Vitvi15g01204\_t001 |  |  |  |  |  |  |  |  |  |  |  |  |
| 0 | Vitvi15g04324\_t001 |  |  |  |  |  |  |  |  |  |  |  |  |
| 0 | Vitvi15g04325\_t001 |  |  |  |  |  |  |  |  |  |  |  |  |
| 0 | Vitvi15g04326\_t001 |  |  |  |  |  |  |  |  |  |  |  |  |
| 0 | Vitvi15g01203\_t001 |  |  |  |  |  |  |  |  |  |  |  |  |
| 0 | Vitvi15g01426\_t001 |  |  |  |  |  |  |  |  |  |  |  |  |
| 0 | Vitvi15g01427\_t001 |  |  |  |  |  |  |  |  |  |  |  |  |
| 0 | Vitvi15g00567\_t001 |  |  |  |  |  |  |  |  |  |  |  |  |
| 0 | Vitvi15g04327\_t001 |  |  |  |  |  |  |  |  |  |  |  |  |
| 0 | Vitvi15g04328\_t001 |  |  |  |  |  |  |  |  |  |  |  |  |
| 0 | Vitvi15g00568\_t001 |  |  |  |  |  |  |  |  |  |  |  |  |
| 0 | Vitvi15g01431\_t001 |  |  |  |  |  |  |  |  |  |  |  |  |
| 0 | Vitvi15g04329\_t001 |  |  |  |  |  |  |  |  |  |  |  |  |
| 0 | Vitvi15g01434\_t002 |  |  |  |  |  |  |  |  |  |  |  |  |
| 0 | Vitvi15g04330\_t001 |  |  |  |  |  |  |  |  |  |  |  |  |
| 0 | Vitvi15g04331\_t001 |  |  |  |  |  |  |  |  |  |  |  |  |
| 0 | Vitvi15g00570\_t001 |  |  |  |  |  |  |  |  |  |  |  |  |
| 0 | Vitvi15g00571\_t001 |  |  |  |  |  |  |  |  |  |  |  |  |
| 0 | Vitvi15g04332\_t001 |  |  |  |  |  |  |  |  |  |  |  |  |
| 0 | Vitvi15g04333\_t001 |  |  |  |  |  |  |  |  |  |  |  |  |
| 0 | Vitvi15g04334\_t001 |  |  |  |  |  |  |  |  |  |  |  |  |
| 0 | Vitvi15g04335\_t001 |  |  |  |  |  |  |  |  |  |  |  |  |
| 0 | Vitvi15g00572\_t001 |  |  |  |  |  |  |  |  |  |  |  |  |
| 0 | Vitvi15g04336\_t001 |  |  |  |  |  |  |  |  |  |  |  |  |
| 0 | Vitvi15g00573\_t001 |  |  |  |  |  |  |  |  |  |  |  |  |
| 0 | Vitvi15g00574\_t001 |  |  |  |  |  |  |  |  |  |  |  |  |
| 0 | Vitvi15g00575\_t001 |  |  |  |  |  |  |  |  |  |  |  |  |
| 0 | Vitvi15g04337\_t001 |  |  |  |  |  |  |  |  |  |  |  |  |
| 0 | Vitvi15g00577\_t001 |  |  |  |  |  |  |  |  |  |  |  |  |
| 0 | Vitvi15g00578\_t002 |  |  |  |  |  |  |  |  |  |  |  |  |
| 0 | Vitvi15g00579\_t001 |  |  |  |  |  |  |  |  |  |  |  |  |
| 0 | Vitvi15g00581\_t001 |  |  |  |  |  |  |  |  |  |  |  |  |
| 0 | Vitvi15g00582\_t001 |  |  |  |  |  |  |  |  |  |  |  |  |
| 0 | Vitvi15g04338\_t001 |  |  |  |  |  |  |  |  |  |  |  |  |
| 0 | Vitvi15g04339\_t001 |  |  |  |  |  |  |  |  |  |  |  |  |
| 0 | Vitvi15g00584\_t001 |  |  |  |  |  |  |  |  |  |  |  |  |
| 0 | Vitvi15g00585\_t001 |  |  |  |  |  |  |  |  |  |  |  |  |
| 0 | Vitvi15g01445\_t001 |  |  |  |  |  |  |  |  |  |  |  |  |
| 0 | Vitvi15g01446\_t001 |  |  |  |  |  |  |  |  |  |  |  |  |
| 0 | Vitvi15g04340\_t001 |  |  |  |  |  |  |  |  |  |  |  |  |
| 0 | Vitvi15g00590\_t001 |  |  |  |  |  |  |  |  |  |  |  |  |
| 0 | Vitvi15g04341\_t001 |  |  |  |  |  |  |  |  |  |  |  |  |
| 0 | Vitvi15g04342\_t001 |  |  |  |  |  |  |  |  |  |  |  |  |
| 0 | Vitvi15g01452\_t001 |  |  |  |  |  |  |  |  |  |  |  |  |
| 0 | Vitvi15g04343\_t001 |  |  |  |  |  |  |  |  |  |  |  |  |
| 0 | Vitvi15g01453\_t001 |  |  |  |  |  |  |  |  |  |  |  |  |
| 0 | Vitvi15g04344\_t001 |  |  |  |  |  |  |  |  |  |  |  |  |
| 0 | Vitvi15g04345\_t001 |  |  |  |  |  |  |  |  |  |  |  |  |
| 0 | Vitvi15g04346\_t001 |  |  |  |  |  |  |  |  |  |  |  |  |
| 0 | Vitvi15g00593\_t001 |  |  |  |  |  |  |  |  |  |  |  |  |
| 0 | Vitvi15g01454\_t001 |  |  |  |  |  |  |  |  |  |  |  |  |
| 0 | Vitvi15g00594\_t001 |  |  |  |  |  |  |  |  |  |  |  |  |
| 0 | Vitvi15g00595\_t001 |  |  |  |  |  |  |  |  |  |  |  |  |
| 0 | Vitvi15g04347\_t001 |  |  |  |  |  |  |  |  |  |  |  |  |
| 0 | Vitvi15g04348\_t001 |  |  |  |  |  |  |  |  |  |  |  |  |
| 0 | Vitvi15g04349\_t001 |  |  |  |  |  |  |  |  |  |  |  |  |
| 0 | Vitvi15g04350\_t001 |  |  |  |  |  |  |  |  |  |  |  |  |
| 0 | Vitvi15g00597\_t001 |  |  |  |  |  |  |  |  |  |  |  |  |
| 0 | Vitvi15g04351\_t001 |  |  |  |  |  |  |  |  |  |  |  |  |
| 0 | Vitvi15g01455\_t001 |  |  |  |  |  |  |  |  |  |  |  |  |
| 0 | Vitvi15g04352\_t001 |  |  |  |  |  |  |  |  |  |  |  |  |
| 0 | Vitvi15g00600\_t001 |  |  |  |  |  |  |  |  |  |  |  |  |
| 0 | Vitvi15g00601\_t001 |  |  |  |  |  |  |  |  |  |  |  |  |
| 0 | Vitvi15g04353\_t001 |  |  |  |  |  |  |  |  |  |  |  |  |
| 0 | Vitvi15g00604\_t004 |  |  |  |  |  |  |  |  |  |  |  |  |
| 0 | Vitvi15g00606\_t001 |  |  |  |  |  |  |  |  |  |  |  |  |
| 0 | Vitvi15g00607\_t001 |  |  |  |  |  |  |  |  |  |  |  |  |
| 0 | Vitvi15g04354\_t001 |  |  |  |  |  |  |  |  |  |  |  |  |
| 0 | Vitvi15g04355\_t001 |  |  |  |  |  |  |  |  |  |  |  |  |
| 0 | Vitvi15g00610\_t001 |  |  |  |  |  |  |  |  |  |  |  |  |
| 0 | Vitvi15g04356\_t001 |  |  |  |  |  |  |  |  |  |  |  |  |
| 0 | Vitvi15g00611\_t001 |  |  |  |  |  |  |  |  |  |  |  |  |
| 0 | Vitvi15g00612\_t001 |  |  |  |  |  |  |  |  |  |  |  |  |
| 0 | Vitvi15g00613\_t001 |  |  |  |  |  |  |  |  |  |  |  |  |
| 0 | Vitvi15g01459\_t001 |  |  |  |  |  |  |  |  |  |  |  |  |
| 0 | Vitvi15g01211\_t001 |  |  |  |  |  |  |  |  |  |  |  |  |
| 0 | Vitvi15g00614\_t001 |  |  |  |  |  |  |  |  |  |  |  |  |
| 0 | Vitvi15g00615\_t001 |  |  |  |  |  |  |  |  |  |  |  |  |
| 0 | Vitvi15g01212\_t001 |  |  |  |  |  |  |  |  |  |  |  |  |
| 0 | Vitvi15g00616\_t001 |  |  |  |  |  |  |  |  |  |  |  |  |
| 0 | Vitvi15g00617\_t001 |  |  |  |  |  |  |  |  |  |  |  |  |
| 0 | Vitvi15g00618\_t001 |  |  |  |  |  |  |  |  |  |  |  |  |
| 0 | Vitvi15g00619\_t001 |  |  |  |  |  |  |  |  |  |  |  |  |
| 0 | Vitvi15g00620\_t001 |  |  |  |  |  |  |  |  |  |  |  |  |
| 0 | Vitvi15g00621\_t001 |  |  |  |  |  |  |  |  |  |  |  |  |
| 0 | Vitvi15g04357\_t001 |  |  |  |  |  |  |  |  |  |  |  |  |
| 0 | Vitvi15g04358\_t001 |  |  |  |  |  |  |  |  |  |  |  |  |
| 0 | Vitvi15g04359\_t001 |  |  |  |  |  |  |  |  |  |  |  |  |
| 0 | Vitvi15g04360\_t001 |  |  |  |  |  |  |  |  |  |  |  |  |
| 0 | Vitvi15g04361\_t001 |  |  |  |  |  |  |  |  |  |  |  |  |
| 0 | Vitvi15g04362\_t001 |  |  |  |  |  |  |  |  |  |  |  |  |
| 0 | Vitvi15g04363\_t001 |  |  |  |  |  |  |  |  |  |  |  |  |
| 0 | Vitvi15g04364\_t001 |  |  |  |  |  |  |  |  |  |  |  |  |
| 0 | Vitvi15g04365\_t001 |  |  |  |  |  |  |  |  |  |  |  |  |
| 0 | Vitvi15g00627\_t001 |  |  |  |  |  |  |  |  |  |  |  |  |
| 0 | Vitvi15g04366\_t001 |  |  |  |  |  |  |  |  |  |  |  |  |
| 0 | Vitvi15g04367\_t001 |  |  |  |  |  |  |  |  |  |  |  |  |
| 0 | Vitvi15g00628\_t002 |  |  |  |  |  |  |  |  |  |  |  |  |
| 0 | Vitvi15g00629\_t001 |  |  |  |  |  |  |  |  |  |  |  |  |
| 0 | Vitvi15g04368\_t001 |  |  |  |  |  |  |  |  |  |  |  |  |
| 0 | Vitvi15g00632\_t001 |  |  |  |  |  |  |  |  |  |  |  |  |
| 0 | Vitvi15g00633\_t001 |  |  |  |  |  |  |  |  |  |  |  |  |
| 0 | Vitvi15g01467\_t001 |  |  |  |  |  |  |  |  |  |  |  |  |
| 0 | Vitvi15g00634\_t001 |  |  |  |  |  |  |  |  |  |  |  |  |
| 0 | Vitvi15g01468\_t001 |  |  |  |  |  |  |  |  |  |  |  |  |
| 0 | Vitvi15g00636\_t001 |  |  |  |  |  |  |  |  |  |  |  |  |
| 0 | Vitvi15g01469\_t001 |  |  |  |  |  |  |  |  |  |  |  |  |
| 0 | Vitvi15g00637\_t001 |  |  |  |  |  |  |  |  |  |  |  |  |
| 0 | Vitvi15g01470\_t001 |  |  |  |  |  |  |  |  |  |  |  |  |
| 0 | Vitvi15g01471\_t001 |  |  |  |  |  |  |  |  |  |  |  |  |
| 0 | Vitvi15g04369\_t001 |  |  |  |  |  |  |  |  |  |  |  |  |
| 0 | Vitvi15g04370\_t001 |  |  |  |  |  |  |  |  |  |  |  |  |
| 0 | Vitvi15g04371\_t001 |  |  |  |  |  |  |  |  |  |  |  |  |
| 0 | Vitvi15g04372\_t001 |  |  |  |  |  |  |  |  |  |  |  |  |
| 0 | Vitvi15g04373\_t001 |  |  |  |  |  |  |  |  |  |  |  |  |
| 0 | Vitvi15g00638\_t001 |  |  |  |  |  |  |  |  |  |  |  |  |
| 0 | Vitvi15g04374\_t001 |  |  |  |  |  |  |  |  |  |  |  |  |
| 0 | Vitvi15g00639\_t001 |  |  |  |  |  |  |  |  |  |  |  |  |
| 0 | Vitvi15g00640\_t001 |  |  |  |  |  |  |  |  |  |  |  |  |
| 0 | Vitvi15g00641\_t001 |  |  |  |  |  |  |  |  |  |  |  |  |
| 0 | Vitvi15g00642\_t001 |  |  |  |  |  |  |  |  |  |  |  |  |
| 0 | Vitvi15g04375\_t001 |  |  |  |  |  |  |  |  |  |  |  |  |
| 0 | Vitvi15g04376\_t001 |  |  |  |  |  |  |  |  |  |  |  |  |
| 0 | Vitvi15g00643\_t001 |  |  |  |  |  |  |  |  |  |  |  |  |
| 0 | Vitvi15g00644\_t001 |  |  |  |  |  |  |  |  |  |  |  |  |
| 0 | Vitvi15g01472\_t001 |  |  |  |  |  |  |  |  |  |  |  |  |
| 0 | Vitvi15g00646\_t001 |  |  |  |  |  |  |  |  |  |  |  |  |
| 0 | Vitvi15g00647\_t001 |  |  |  |  |  |  |  |  |  |  |  |  |
| 0 | Vitvi15g00648\_t001 |  |  |  |  |  |  |  |  |  |  |  |  |
| 0 | Vitvi15g00650\_t001 |  |  |  |  |  |  |  |  |  |  |  |  |
| 0 | Vitvi15g00653\_t001 |  |  |  |  |  |  |  |  |  |  |  |  |
| 0 | Vitvi15g01473\_t001 |  |  |  |  |  |  |  |  |  |  |  |  |
| 0 | Vitvi15g04377\_t001 |  |  |  |  |  |  |  |  |  |  |  |  |
| 0 | Vitvi15g04378\_t001 |  |  |  |  |  |  |  |  |  |  |  |  |
| 0 | Vitvi15g04379\_t001 |  |  |  |  |  |  |  |  |  |  |  |  |
| 0 | Vitvi15g04380\_t001 |  |  |  |  |  |  |  |  |  |  |  |  |
| 0 | Vitvi15g04381\_t001 |  |  |  |  |  |  |  |  |  |  |  |  |
| 0 | Vitvi15g04382\_t001 |  |  |  |  |  |  |  |  |  |  |  |  |
| 0 | Vitvi15g04383\_t001 |  |  |  |  |  |  |  |  |  |  |  |  |
| 0 | Vitvi15g01476\_t001 |  |  |  |  |  |  |  |  |  |  |  |  |
| 0 | Vitvi15g04384\_t001 |  |  |  |  |  |  |  |  |  |  |  |  |
| 0 | Vitvi15g01477\_t001 |  |  |  |  |  |  |  |  |  |  |  |  |
| 0 | Vitvi15g04386\_t001 |  |  |  |  |  |  |  |  |  |  |  |  |
| 0 | Vitvi15g04387\_t001 |  |  |  |  |  |  |  |  |  |  |  |  |
| 0 | Vitvi15g04388\_t001 |  |  |  |  |  |  |  |  |  |  |  |  |
| 0 | Vitvi15g04389\_t001 |  |  |  |  |  |  |  |  |  |  |  |  |
| 0 | Vitvi15g04390\_t001 |  |  |  |  |  |  |  |  |  |  |  |  |
| 0 | Vitvi15g04391\_t001 |  |  |  |  |  |  |  |  |  |  |  |  |
| 0 | Vitvi15g01480\_t001 |  |  |  |  |  |  |  |  |  |  |  |  |
| 0 | Vitvi15g00661\_t001 |  |  |  |  |  |  |  |  |  |  |  |  |
| 0 | Vitvi15g04392\_t001 |  |  |  |  |  |  |  |  |  |  |  |  |
| 0 | Vitvi15g04393\_t001 |  |  |  |  |  |  |  |  |  |  |  |  |
| 0 | Vitvi15g04394\_t001 |  |  |  |  |  |  |  |  |  |  |  |  |
| 0 | Vitvi15g04395\_t001 |  |  |  |  |  |  |  |  |  |  |  |  |
| 0 | Vitvi15g04396\_t001 |  |  |  |  |  |  |  |  |  |  |  |  |
| 0 | Vitvi15g04397\_t001 |  |  |  |  |  |  |  |  |  |  |  |  |
| 0 | Vitvi15g04398\_t001 |  |  |  |  |  |  |  |  |  |  |  |  |
| 0 | Vitvi15g04399\_t001 |  |  |  |  |  |  |  |  |  |  |  |  |
| 0 | Vitvi15g04400\_t001 |  |  |  |  |  |  |  |  |  |  |  |  |
| 0 | Vitvi15g04401\_t001 |  |  |  |  |  |  |  |  |  |  |  |  |
| 0 | Vitvi15g04402\_t001 |  |  |  |  |  |  |  |  |  |  |  |  |
| 0 | Vitvi15g04403\_t001 |  |  |  |  |  |  |  |  |  |  |  |  |
| 0 | Vitvi15g04404\_t001 |  |  |  |  |  |  |  |  |  |  |  |  |
| 0 | Vitvi15g04405\_t001 |  |  |  |  |  |  |  |  |  |  |  |  |
| 0 | Vitvi15g04406\_t001 |  |  |  |  |  |  |  |  |  |  |  |  |
| 0 | Vitvi15g04407\_t001 |  |  |  |  |  |  |  |  |  |  |  |  |
| 0 | Vitvi15g04408\_t001 |  |  |  |  |  |  |  |  |  |  |  |  |
| 0 | Vitvi15g01483\_t001 |  |  |  |  |  |  |  |  |  |  |  |  |
| 0 | Vitvi15g00670\_t001 |  |  |  |  |  |  |  |  |  |  |  |  |
| 0 | Vitvi15g04409\_t001 |  |  |  |  |  |  |  |  |  |  |  |  |
| 0 | Vitvi15g04410\_t001 |  |  |  |  |  |  |  |  |  |  |  |  |
| 0 | Vitvi15g04411\_t001 |  |  |  |  |  |  |  |  |  |  |  |  |
| 0 | Vitvi15g04412\_t001 |  |  |  |  |  |  |  |  |  |  |  |  |
| 0 | Vitvi15g04413\_t001 |  |  |  |  |  |  |  |  |  |  |  |  |
| 0 | Vitvi15g04414\_t001 |  |  |  |  |  |  |  |  |  |  |  |  |
| 0 | Vitvi15g00674\_t001 |  |  |  |  |  |  |  |  |  |  |  |  |
| 0 | Vitvi15g00676\_t001 |  |  |  |  |  |  |  |  |  |  |  |  |
| 0 | Vitvi15g00677\_t001 |  |  |  |  |  |  |  |  |  |  |  |  |
| 0 | Vitvi15g00678\_t001 |  |  |  |  |  |  |  |  |  |  |  |  |
| 0 | Vitvi15g00679\_t001 |  |  |  |  |  |  |  |  |  |  |  |  |
| 0 | Vitvi15g00680\_t001 |  |  |  |  |  |  |  |  |  |  |  |  |
| 0 | Vitvi15g01488\_t002 |  |  |  |  |  |  |  |  |  |  |  |  |
| 0 | Vitvi15g04415\_t001 |  |  |  |  |  |  |  |  |  |  |  |  |
| 0 | Vitvi15g00682\_t001 |  |  |  |  |  |  |  |  |  |  |  |  |
| 1 | Vitvi15g00684\_t001 |  | Vitvi02g04132\_t001 |  |  |  |  |  |  |  |  |  |  |
| 1 | Vitvi15g01489\_t001 |  | \| \| |  |  |  |  |  |  |  |  |  |  |
| 1 | Vitvi15g00687\_t001 |  | \| \| |  |  |  |  |  |  |  |  |  |  |
| 1 | Vitvi15g01490\_t001 |  | \| \| |  |  |  |  |  |  |  |  |  |  |
| 1 | Vitvi15g01491\_t001 |  | \| \| |  |  |  |  |  |  |  |  |  |  |
| 1 | Vitvi15g04416\_t001 |  | \| \| |  |  |  |  |  |  |  |  |  |  |
| 1 | Vitvi15g00689\_t001 |  | \| \| |  |  |  |  |  |  |  |  |  |  |
| 1 | Vitvi15g00690\_t001 |  | \| \| |  |  |  |  |  |  |  |  |  |  |
| 1 | Vitvi15g00691\_t001 |  | \| \| |  |  |  |  |  |  |  |  |  |  |
| 1 | Vitvi15g00692\_t001 |  | \| \| |  |  |  |  |  |  |  |  |  |  |
| 1 | Vitvi15g00693\_t001 |  | \| \| |  |  |  |  |  |  |  |  |  |  |
| 1 | Vitvi15g00694\_t001 |  | \| \| |  |  |  |  |  |  |  |  |  |  |
| 1 | Vitvi15g00695\_t001 |  | \| \| |  |  |  |  |  |  |  |  |  |  |
| 1 | Vitvi15g00696\_t001 |  | \| \| |  |  |  |  |  |  |  |  |  |  |
| 1 | Vitvi15g04417\_t001 |  | \| \| |  |  |  |  |  |  |  |  |  |  |
| 1 | Vitvi15g00697\_t001 |  | \| \| |  |  |  |  |  |  |  |  |  |  |
| 1 | Vitvi15g00698\_t001 |  | Vitvi02g00499\_t001 |  |  |  |  |  |  |  |  |  |  |
| 1 | Vitvi15g00699\_t001 |  | \| \| |  |  |  |  |  |  |  |  |  |  |
| 1 | Vitvi15g00701\_t001 |  | \| \| |  |  |  |  |  |  |  |  |  |  |
| 1 | Vitvi15g01207\_t001 |  | \| \| |  |  |  |  |  |  |  |  |  |  |
| 1 | Vitvi15g00702\_t001 |  | \| \| |  |  |  |  |  |  |  |  |  |  |
| 1 | Vitvi15g01208\_t001 |  | \| \| |  |  |  |  |  |  |  |  |  |  |
| 1 | Vitvi15g00703\_t001 |  | \| \| |  |  |  |  |  |  |  |  |  |  |
| 1 | Vitvi15g00704\_t001 |  | \| \| |  |  |  |  |  |  |  |  |  |  |
| 1 | Vitvi15g00705\_t001 |  | \| \| |  |  |  |  |  |  |  |  |  |  |
| 1 | Vitvi15g00706\_t001 |  | Vitvi02g00507\_t001 |  |  |  |  |  |  |  |  |  |  |
| 1 | Vitvi15g04418\_t001 |  | \| \| |  |  |  |  |  |  |  |  |  |  |
| 1 | Vitvi15g00708\_t002 |  | Vitvi02g00510\_t001 |  |  |  |  |  |  |  |  |  |  |
| 1 | Vitvi15g00709\_t001 |  | \| \| |  |  |  |  |  |  |  |  |  |  |
| 1 | Vitvi15g04419\_t001 |  | \| \| |  |  |  |  |  |  |  |  |  |  |
| 1 | Vitvi15g00710\_t001 |  | Vitvi02g01691\_t001 |  |  |  |  |  |  |  |  |  |  |
| 1 | Vitvi15g04420\_t001 |  | \| \| |  |  |  |  |  |  |  |  |  |  |
| 1 | Vitvi15g00711\_t001 |  | \| \| |  |  |  |  |  |  |  |  |  |  |
| 1 | Vitvi15g04421\_t001 |  | \| \| |  |  |  |  |  |  |  |  |  |  |
| 1 | Vitvi15g04422\_t001 |  | \| \| |  |  |  |  |  |  |  |  |  |  |
| 1 | Vitvi15g00713\_t001 |  | \| \| |  |  |  |  |  |  |  |  |  |  |
| 1 | Vitvi15g01494\_t001 |  | \| \| |  |  |  |  |  |  |  |  |  |  |
| 1 | Vitvi15g01495\_t001 |  | \| \| |  |  |  |  |  |  |  |  |  |  |
| 1 | Vitvi15g00714\_t001 |  | Vitvi02g01438\_t001 |  |  |  |  |  |  |  |  |  |  |
| 1 | Vitvi15g00715\_t001 |  | Vitvi02g00533\_t001 |  |  |  |  |  |  |  |  |  |  |
| 1 | Vitvi15g00716\_t001 |  | \| \| |  |  |  |  |  |  |  |  |  |  |
| 1 | Vitvi15g01496\_t001 |  | \| \| |  |  |  |  |  |  |  |  |  |  |
| 1 | Vitvi15g00718\_t002 |  | Vitvi02g00535\_t001 |  |  |  |  |  |  |  |  |  |  |
| 0 | Vitvi15g00719\_t001 |  |  |  |  |  |  |  |  |  |  |  |  |
| 0 | Vitvi15g00721\_t001 |  |  |  |  |  |  |  |  |  |  |  |  |
| 0 | Vitvi15g00723\_t001 |  |  |  |  |  |  |  |  |  |  |  |  |
| 0 | Vitvi15g04423\_t001 |  |  |  |  |  |  |  |  |  |  |  |  |
| 0 | Vitvi15g00724\_t001 |  |  |  |  |  |  |  |  |  |  |  |  |
| 0 | Vitvi15g00725\_t001 |  |  |  |  |  |  |  |  |  |  |  |  |
| 0 | Vitvi15g00726\_t002 |  |  |  |  |  |  |  |  |  |  |  |  |
| 1 | Vitvi15g00727\_t001 |  | Vitvi02g00460\_t002 |  |  |  |  |  |  |  |  |  |  |
| 1 | Vitvi15g00728\_t002 |  | \| \| |  |  |  |  |  |  |  |  |  |  |
| 1 | Vitvi15g00731\_t001 |  | Vitvi02g00462\_t001 |  |  |  |  |  |  |  |  |  |  |
| 1 | Vitvi15g00732\_t001 |  | Vitvi02g00465\_t001 |  |  |  |  |  |  |  |  |  |  |
| 1 | Vitvi15g01499\_t001 |  | \| \| |  |  |  |  |  |  |  |  |  |  |
| 1 | Vitvi15g00733\_t001 |  | \| \| |  |  |  |  |  |  |  |  |  |  |
| 1 | Vitvi15g00734\_t001 |  | \| \| |  |  |  |  |  |  |  |  |  |  |
| 1 | Vitvi15g00735\_t001 |  | \| \| |  |  |  |  |  |  |  |  |  |  |
| 1 | Vitvi15g00736\_t001 |  | \| \| |  |  |  |  |  |  |  |  |  |  |
| 1 | Vitvi15g00737\_t001 |  | \| \| |  |  |  |  |  |  |  |  |  |  |
| 1 | Vitvi15g04424\_t001 |  | \| \| |  |  |  |  |  |  |  |  |  |  |
| 1 | Vitvi15g04425\_t001 |  | \| \| |  |  |  |  |  |  |  |  |  |  |
| 1 | Vitvi15g04426\_t001 |  | \| \| |  |  |  |  |  |  |  |  |  |  |
| 1 | Vitvi15g00739\_t001 |  | \| \| |  |  |  |  |  |  |  |  |  |  |
| 1 | Vitvi15g00740\_t001 |  | Vitvi02g00468\_t001 |  |  |  |  |  |  |  |  |  |  |
| 1 | Vitvi15g01500\_t001 |  | Vitvi02g04123\_t001 |  |  |  |  |  |  |  |  |  |  |
| 1 | Vitvi15g00741\_t001 |  | Vitvi02g00471\_t001 |  |  |  |  |  |  |  |  |  |  |
| 0 | Vitvi15g00742\_t001 |  |  |  |  |  |  |  |  |  |  |  |  |
| 0 | Vitvi15g00743\_t001 |  |  |  |  |  |  |  |  |  |  |  |  |
| 0 | Vitvi15g00744\_t001 |  |  |  |  |  |  |  |  |  |  |  |  |
| 0 | Vitvi15g00745\_t001 |  |  |  |  |  |  |  |  |  |  |  |  |
| 0 | Vitvi15g00746\_t001 |  |  |  |  |  |  |  |  |  |  |  |  |
| 0 | Vitvi15g00747\_t001 |  |  |  |  |  |  |  |  |  |  |  |  |
| 0 | Vitvi15g04427\_t001 |  |  |  |  |  |  |  |  |  |  |  |  |
| 0 | Vitvi15g00748\_t001 |  |  |  |  |  |  |  |  |  |  |  |  |
| 0 | Vitvi15g01501\_t001 |  |  |  |  |  |  |  |  |  |  |  |  |
| 0 | Vitvi15g01503\_t001 |  |  |  |  |  |  |  |  |  |  |  |  |
| 0 | Vitvi15g01504\_t001 |  |  |  |  |  |  |  |  |  |  |  |  |
| 1 | Vitvi15g00749\_t001 |  | Vitvi16g00947\_t001 |  |  |  |  |  |  |  |  |  |  |
| 1 | Vitvi15g00751\_t001 |  | \| \| |  |  |  |  |  |  |  |  |  |  |
| 1 | Vitvi15g00752\_t001 |  | \| \| |  |  |  |  |  |  |  |  |  |  |
| 2 | Vitvi15g00753\_t001 |  | \| \| |  | Vitvi02g00409\_t001 |  |  |  |  |  |  |  |  |
| 2 | Vitvi15g00754\_t001 |  | \| \| |  | Vitvi02g00410\_t001 |  |  |  |  |  |  |  |  |
| 2 | Vitvi15g00757\_t001 |  | \| \| |  | \| \| |  |  |  |  |  |  |  |  |
| 2 | Vitvi15g00758\_t001 |  | \| \| |  | \| \| |  |  |  |  |  |  |  |  |
| 2 | Vitvi15g01505\_t001 |  | Vitvi16g00926\_t001 |  | \| \| |  |  |  |  |  |  |  |  |
| 2 | Vitvi15g00759\_t001 |  | \| \| |  | Vitvi02g00415\_t001 |  |  |  |  |  |  |  |  |
| 2 | Vitvi15g00760\_t001 |  | \| \| |  | \| \| |  |  |  |  |  |  |  |  |
| 2 | Vitvi15g00761\_t001 |  | \| \| |  | \| \| |  |  |  |  |  |  |  |  |
| 2 | Vitvi15g00762\_t001 |  | \| \| |  | \| \| |  |  |  |  |  |  |  |  |
| 2 | Vitvi15g00763\_t001 |  | \| \| |  | \| \| |  |  |  |  |  |  |  |  |
| 2 | Vitvi15g00764\_t001 |  | \| \| |  | \| \| |  |  |  |  |  |  |  |  |
| 2 | Vitvi15g00765\_t001 |  | Vitvi16g01846\_t001 |  | Vitvi02g00421\_t001 |  |  |  |  |  |  |  |  |
| 2 | Vitvi15g00766\_t001 |  | \| \| |  | Vitvi02g00424\_t001 |  |  |  |  |  |  |  |  |
| 2 | Vitvi15g00767\_t001 |  | \| \| |  | \| \| |  |  |  |  |  |  |  |  |
| 2 | Vitvi15g04428\_t001 |  | \| \| |  | \| \| |  |  |  |  |  |  |  |  |
| 2 | Vitvi15g01506\_t001 |  | \| \| |  | \| \| |  |  |  |  |  |  |  |  |
| 2 | Vitvi15g00768\_t001 |  | \| \| |  | \| \| |  |  |  |  |  |  |  |  |
| 2 | Vitvi15g00769\_t001 |  | \| \| |  | \| \| |  |  |  |  |  |  |  |  |
| 2 | Vitvi15g00770\_t001 |  | \| \| |  | \| \| |  |  |  |  |  |  |  |  |
| 2 | Vitvi15g00771\_t001 |  | \| \| |  | \| \| |  |  |  |  |  |  |  |  |
| 2 | Vitvi15g00772\_t001 |  | \| \| |  | \| \| |  |  |  |  |  |  |  |  |
| 2 | Vitvi15g04429\_t001 |  | \| \| |  | \| \| |  |  |  |  |  |  |  |  |
| 2 | Vitvi15g00773\_t002 |  | \| \| |  | \| \| |  |  |  |  |  |  |  |  |
| 2 | Vitvi15g00774\_t002 |  | Vitvi16g00898\_t001 |  | Vitvi02g00427\_t001 |  |  |  |  |  |  |  |  |
| 2 | Vitvi15g00775\_t001 |  | Vitvi16g00895\_t001 |  | \| \| |  |  |  |  |  |  |  |  |
| 2 | Vitvi15g00776\_t001 |  | Vitvi16g00894\_t001 |  | \| \| |  |  |  |  |  |  |  |  |
| 2 | Vitvi15g00781\_t003 |  | \| \| |  | \| \| |  |  |  |  |  |  |  |  |
| 2 | Vitvi15g00782\_t001 |  | Vitvi16g00890\_t001 |  | \| \| |  |  |  |  |  |  |  |  |
| 2 | Vitvi15g00783\_t002 |  | \| \| |  | Vitvi02g00436\_t001 |  |  |  |  |  |  |  |  |
| 1 | Vitvi15g00785\_t001 |  | \| \| |  |  |  |  |  |  |  |  |  |  |
| 1 | Vitvi15g00786\_t001 |  | \| \| |  |  |  |  |  |  |  |  |  |  |
| 1 | Vitvi15g01508\_t001 |  | Vitvi16g00889\_t001 |  |  |  |  |  |  |  |  |  |  |
| 1 | Vitvi15g00787\_t001 |  | \| \| |  |  |  |  |  |  |  |  |  |  |
| 1 | Vitvi15g00789\_t001 |  | \| \| |  |  |  |  |  |  |  |  |  |  |
| 1 | Vitvi15g04430\_t001 |  | \| \| |  |  |  |  |  |  |  |  |  |  |
| 1 | Vitvi15g04431\_t001 |  | \| \| |  |  |  |  |  |  |  |  |  |  |
| 1 | Vitvi15g00790\_t001 |  | Vitvi16g00880\_t001 |  |  |  |  |  |  |  |  |  |  |
| 0 | Vitvi15g00791\_t002 |  |  |  |  |  |  |  |  |  |  |  |  |
| 0 | Vitvi15g00792\_t001 |  |  |  |  |  |  |  |  |  |  |  |  |
| 0 | Vitvi15g04432\_t001 |  |  |  |  |  |  |  |  |  |  |  |  |
| 0 | Vitvi15g04433\_t001 |  |  |  |  |  |  |  |  |  |  |  |  |
| 0 | Vitvi15g04434\_t001 |  |  |  |  |  |  |  |  |  |  |  |  |
| 0 | Vitvi15g04435\_t001 |  |  |  |  |  |  |  |  |  |  |  |  |
| 0 | Vitvi15g01511\_t001 |  |  |  |  |  |  |  |  |  |  |  |  |
| 0 | Vitvi15g01512\_t001 |  |  |  |  |  |  |  |  |  |  |  |  |
| 0 | Vitvi15g04436\_t001 |  |  |  |  |  |  |  |  |  |  |  |  |
| 0 | Vitvi15g00795\_t001 |  |  |  |  |  |  |  |  |  |  |  |  |
| 0 | Vitvi15g04437\_t001 |  |  |  |  |  |  |  |  |  |  |  |  |
| 0 | Vitvi15g00796\_t001 |  |  |  |  |  |  |  |  |  |  |  |  |
| 0 | Vitvi15g00798\_t001 |  |  |  |  |  |  |  |  |  |  |  |  |
| 0 | Vitvi15g04438\_t001 |  |  |  |  |  |  |  |  |  |  |  |  |
| 0 | Vitvi15g04439\_t001 |  |  |  |  |  |  |  |  |  |  |  |  |
| 0 | Vitvi15g01514\_t001 |  |  |  |  |  |  |  |  |  |  |  |  |
| 0 | Vitvi15g04440\_t001 |  |  |  |  |  |  |  |  |  |  |  |  |
| 0 | Vitvi15g04441\_t001 |  |  |  |  |  |  |  |  |  |  |  |  |
| 0 | Vitvi15g00801\_t001 |  |  |  |  |  |  |  |  |  |  |  |  |
| 0 | Vitvi15g00803\_t001 |  |  |  |  |  |  |  |  |  |  |  |  |
| 0 | Vitvi15g00804\_t003 |  |  |  |  |  |  |  |  |  |  |  |  |
| 0 | Vitvi15g04442\_t001 |  |  |  |  |  |  |  |  |  |  |  |  |
| 0 | Vitvi15g01518\_t001 |  |  |  |  |  |  |  |  |  |  |  |  |
| 0 | Vitvi15g00805\_t001 |  |  |  |  |  |  |  |  |  |  |  |  |
| 0 | Vitvi15g04443\_t001 |  |  |  |  |  |  |  |  |  |  |  |  |
| 0 | Vitvi15g04444\_t001 |  |  |  |  |  |  |  |  |  |  |  |  |
| 0 | Vitvi15g04445\_t001 |  |  |  |  |  |  |  |  |  |  |  |  |
| 0 | Vitvi15g04446\_t001 |  |  |  |  |  |  |  |  |  |  |  |  |
| 0 | Vitvi15g04447\_t001 |  |  |  |  |  |  |  |  |  |  |  |  |
| 0 | Vitvi15g00807\_t001 |  |  |  |  |  |  |  |  |  |  |  |  |
| 0 | Vitvi15g00808\_t001 |  |  |  |  |  |  |  |  |  |  |  |  |
| 0 | Vitvi15g01519\_t001 |  |  |  |  |  |  |  |  |  |  |  |  |
| 0 | Vitvi15g04448\_t001 |  |  |  |  |  |  |  |  |  |  |  |  |
| 0 | Vitvi15g00810\_t001 |  |  |  |  |  |  |  |  |  |  |  |  |
| 0 | Vitvi15g04449\_t001 |  |  |  |  |  |  |  |  |  |  |  |  |
| 0 | Vitvi15g01524\_t001 |  |  |  |  |  |  |  |  |  |  |  |  |
| 0 | Vitvi15g04450\_t001 |  |  |  |  |  |  |  |  |  |  |  |  |
| 0 | Vitvi15g00811\_t001 |  |  |  |  |  |  |  |  |  |  |  |  |
| 0 | Vitvi15g00812\_t001 |  |  |  |  |  |  |  |  |  |  |  |  |
| 0 | Vitvi15g00813\_t003 |  |  |  |  |  |  |  |  |  |  |  |  |
| 0 | Vitvi15g00814\_t001 |  |  |  |  |  |  |  |  |  |  |  |  |
| 0 | Vitvi15g00815\_t001 |  |  |  |  |  |  |  |  |  |  |  |  |
| 0 | Vitvi15g04451\_t001 |  |  |  |  |  |  |  |  |  |  |  |  |
| 0 | Vitvi15g00816\_t001 |  |  |  |  |  |  |  |  |  |  |  |  |
| 0 | Vitvi15g00817\_t001 |  |  |  |  |  |  |  |  |  |  |  |  |
| 0 | Vitvi15g00818\_t001 |  |  |  |  |  |  |  |  |  |  |  |  |
| 0 | Vitvi15g00819\_t001 |  |  |  |  |  |  |  |  |  |  |  |  |
| 0 | Vitvi15g00820\_t001 |  |  |  |  |  |  |  |  |  |  |  |  |
| 0 | Vitvi15g01525\_t001 |  |  |  |  |  |  |  |  |  |  |  |  |
| 0 | Vitvi15g00821\_t001 |  |  |  |  |  |  |  |  |  |  |  |  |
| 0 | Vitvi15g04452\_t001 |  |  |  |  |  |  |  |  |  |  |  |  |
| 0 | Vitvi15g04453\_t001 |  |  |  |  |  |  |  |  |  |  |  |  |
| 0 | Vitvi15g00823\_t001 |  |  |  |  |  |  |  |  |  |  |  |  |
| 0 | Vitvi15g00824\_t001 |  |  |  |  |  |  |  |  |  |  |  |  |
| 0 | Vitvi15g04454\_t001 |  |  |  |  |  |  |  |  |  |  |  |  |
| 0 | Vitvi15g00825\_t001.2.6037826c |  |  |  |  |  |  |  |  |  |  |  |  |
| 0 | Vitvi15g00826\_t001 |  |  |  |  |  |  |  |  |  |  |  |  |
| 1 | Vitvi15g00827\_t001 |  | Vitvi16g00949\_t004 |  |  |  |  |  |  |  |  |  |  |
| 1 | Vitvi15g00828\_t008 |  | \| \| |  |  |  |  |  |  |  |  |  |  |
| 1 | Vitvi15g00829\_t003 |  | \| \| |  |  |  |  |  |  |  |  |  |  |
| 1 | Vitvi15g00830\_t001 |  | Vitvi16g00956\_t001 |  |  |  |  |  |  |  |  |  |  |
| 1 | Vitvi15g00831\_t002 |  | Vitvi16g01861\_t001 |  |  |  |  |  |  |  |  |  |  |
| 1 | Vitvi15g04455\_t001 |  | \| \| |  |  |  |  |  |  |  |  |  |  |
| 1 | Vitvi15g00832\_t001 |  | Vitvi16g00957\_t001 |  |  |  |  |  |  |  |  |  |  |
| 1 | Vitvi15g00833\_t001 |  | Vitvi16g00964\_t001 |  |  |  |  |  |  |  |  |  |  |
| 1 | Vitvi15g00835\_t001 |  | Vitvi16g00966\_t001 |  |  |  |  |  |  |  |  |  |  |
| 1 | Vitvi15g00837\_t001 |  | \| \| |  |  |  |  |  |  |  |  |  |  |
| 1 | Vitvi15g00838\_t003 |  | \| \| |  |  |  |  |  |  |  |  |  |  |
| 1 | Vitvi15g04456\_t001 |  | \| \| |  |  |  |  |  |  |  |  |  |  |
| 1 | Vitvi15g00839\_t001 |  | Vitvi16g00974\_t001 |  |  |  |  |  |  |  |  |  |  |
| 0 | Vitvi15g00840\_t001 |  |  |  |  |  |  |  |  |  |  |  |  |
| 0 | Vitvi15g04457\_t001 |  |  |  |  |  |  |  |  |  |  |  |  |
| 0 | Vitvi15g00841\_t001 |  |  |  |  |  |  |  |  |  |  |  |  |
| 0 | Vitvi15g00842\_t001 |  |  |  |  |  |  |  |  |  |  |  |  |
| 0 | Vitvi15g04458\_t001 |  |  |  |  |  |  |  |  |  |  |  |  |
| 0 | Vitvi15g01531\_t001 |  |  |  |  |  |  |  |  |  |  |  |  |
| 0 | Vitvi15g04459\_t001 |  |  |  |  |  |  |  |  |  |  |  |  |
| 0 | Vitvi15g00844\_t001 |  |  |  |  |  |  |  |  |  |  |  |  |
| 0 | Vitvi15g00845\_t001.1.6037826b |  |  |  |  |  |  |  |  |  |  |  |  |
| 0 | Vitvi15g00846\_t001 |  |  |  |  |  |  |  |  |  |  |  |  |
| 0 | Vitvi15g01534\_t001 |  |  |  |  |  |  |  |  |  |  |  |  |
| 0 | Vitvi15g01535\_t001 |  |  |  |  |  |  |  |  |  |  |  |  |
| 0 | Vitvi15g00847\_t001 |  |  |  |  |  |  |  |  |  |  |  |  |
| 0 | Vitvi15g04460\_t001 |  |  |  |  |  |  |  |  |  |  |  |  |
| 0 | Vitvi15g04461\_t001 |  |  |  |  |  |  |  |  |  |  |  |  |
| 0 | Vitvi15g04462\_t001 |  |  |  |  |  |  |  |  |  |  |  |  |
| 0 | Vitvi15g00849\_t001 |  |  |  |  |  |  |  |  |  |  |  |  |
| 0 | Vitvi15g00852\_t001 |  |  |  |  |  |  |  |  |  |  |  |  |
| 0 | Vitvi15g00853\_t001 |  |  |  |  |  |  |  |  |  |  |  |  |
| 0 | Vitvi15g00854\_t002 |  |  |  |  |  |  |  |  |  |  |  |  |
| 0 | Vitvi15g00855\_t001 |  |  |  |  |  |  |  |  |  |  |  |  |
| 0 | Vitvi15g00856\_t001 |  |  |  |  |  |  |  |  |  |  |  |  |
| 0 | Vitvi15g00857\_t001 |  |  |  |  |  |  |  |  |  |  |  |  |
| 0 | Vitvi15g01538\_t004 |  |  |  |  |  |  |  |  |  |  |  |  |
| 0 | Vitvi15g00858\_t001 |  |  |  |  |  |  |  |  |  |  |  |  |
| 1 | Vitvi15g00859\_t002 |  | Vitvi16g01326\_t001 |  |  |  |  |  |  |  |  |  |  |
| 1 | Vitvi15g04463\_t001 |  | \| \| |  |  |  |  |  |  |  |  |  |  |
| 2 | Vitvi15g01539\_t001 |  | Vitvi16g01327\_t002 |  | Vitvi02g00277\_t001 |  |  |  |  |  |  |  |  |
| 2 | Vitvi15g01540\_t001 |  | \| \| |  | \| \| |  |  |  |  |  |  |  |  |
| 2 | Vitvi15g01541\_t001 |  | \| \| |  | \| \| |  |  |  |  |  |  |  |  |
| 2 | Vitvi15g01542\_t001 |  | \| \| |  | \| \| |  |  |  |  |  |  |  |  |
| 2 | Vitvi15g01543\_t001 |  | \| \| |  | \| \| |  |  |  |  |  |  |  |  |
| 2 | Vitvi15g01544\_t001 |  | \| \| |  | \| \| |  |  |  |  |  |  |  |  |
| 2 | Vitvi15g04464\_t001 |  | \| \| |  | \| \| |  |  |  |  |  |  |  |  |
| 2 | Vitvi15g04465\_t001 |  | \| \| |  | \| \| |  |  |  |  |  |  |  |  |
| 2 | Vitvi15g01545\_t001 |  | \| \| |  | \| \| |  |  |  |  |  |  |  |  |
| 2 | Vitvi15g04466\_t001 |  | \| \| |  | \| \| |  |  |  |  |  |  |  |  |
| 2 | Vitvi15g01546\_t001 |  | \| \| |  | \| \| |  |  |  |  |  |  |  |  |
| 2 | Vitvi15g04467\_t001 |  | \| \| |  | \| \| |  |  |  |  |  |  |  |  |
| 2 | Vitvi15g00862\_t001 |  | \| \| |  | \| \| |  |  |  |  |  |  |  |  |
| 2 | Vitvi15g01547\_t001 |  | \| \| |  | \| \| |  |  |  |  |  |  |  |  |
| 2 | Vitvi15g00863\_t001 |  | \| \| |  | Vitvi02g00275\_t001 |  |  |  |  |  |  |  |  |
| 2 | Vitvi15g00864\_t001 |  | \| \| |  | \| \| |  |  |  |  |  |  |  |  |
| 2 | Vitvi15g00867\_t001 |  | \| \| |  | Vitvi02g00272\_t001 |  |  |  |  |  |  |  |  |
| 2 | Vitvi15g00868\_t001 |  | \| \| |  | \| \| |  |  |  |  |  |  |  |  |
| 2 | Vitvi15g00869\_t001 |  | \| \| |  | \| \| |  |  |  |  |  |  |  |  |
| 2 | Vitvi15g00870\_t001.1.6037826c |  | \| \| |  | \| \| |  |  |  |  |  |  |  |  |
| 2 | Vitvi15g00871\_t001 |  | Vitvi16g01336\_t001 |  | \| \| |  |  |  |  |  |  |  |  |
| 2 | Vitvi15g00873\_t001 |  | \| \| |  | \| \| |  |  |  |  |  |  |  |  |
| 2 | Vitvi15g00874\_t001 |  | \| \| |  | Vitvi02g00268\_t001 |  |  |  |  |  |  |  |  |
| 2 | Vitvi15g01548\_t001 |  | \| \| |  | \| \| |  |  |  |  |  |  |  |  |
| 2 | Vitvi15g01549\_t001 |  | \| \| |  | \| \| |  |  |  |  |  |  |  |  |
| 2 | Vitvi15g00876\_t001 |  | \| \| |  | \| \| |  |  |  |  |  |  |  |  |
| 2 | Vitvi15g04468\_t001 |  | \| \| |  | \| \| |  |  |  |  |  |  |  |  |
| 2 | Vitvi15g04469\_t001 |  | \| \| |  | \| \| |  |  |  |  |  |  |  |  |
| 2 | Vitvi15g00877\_t001 |  | \| \| |  | Vitvi02g01369\_t001 |  |  |  |  |  |  |  |  |
| 2 | Vitvi15g00879\_t001 |  | Vitvi16g01340\_t001 |  | \| \| |  |  |  |  |  |  |  |  |
| 2 | Vitvi15g00880\_t002 |  | \| \| |  | \| \| |  |  |  |  |  |  |  |  |
| 2 | Vitvi15g00881\_t002 |  | \| \| |  | \| \| |  |  |  |  |  |  |  |  |
| 2 | Vitvi15g00882\_t001 |  | \| \| |  | \| \| |  |  |  |  |  |  |  |  |
| 2 | Vitvi15g00883\_t001 |  | \| \| |  | Vitvi02g00252\_t001 |  |  |  |  |  |  |  |  |
| 2 | Vitvi15g00884\_t001 |  | \| \| |  | \| \| |  |  |  |  |  |  |  |  |
| 2 | Vitvi15g00885\_t001 |  | \| \| |  | Vitvi02g00250\_t001 |  |  |  |  |  |  |  |  |
| 2 | Vitvi15g04470\_t001 |  | Vitvi16g02068\_t001 |  | Vitvi02g00249\_t001 |  |  |  |  |  |  |  |  |
| 2 | Vitvi15g00886\_t001 |  | \| \| |  | \| \| |  |  |  |  |  |  |  |  |
| 2 | Vitvi15g04471\_t001 |  | \| \| |  | \| \| |  |  |  |  |  |  |  |  |
| 2 | Vitvi15g04472\_t001 |  | \| \| |  | \| \| |  |  |  |  |  |  |  |  |
| 2 | Vitvi15g04473\_t001 |  | \| \| |  | \| \| |  |  |  |  |  |  |  |  |
| 2 | Vitvi15g04474\_t001 |  | \| \| |  | \| \| |  |  |  |  |  |  |  |  |
| 2 | Vitvi15g04475\_t001 |  | \| \| |  | \| \| |  |  |  |  |  |  |  |  |
| 2 | Vitvi15g00887\_t001 |  | Vitvi16g02069\_t001 |  | \| \| |  |  |  |  |  |  |  |  |
| 2 | Vitvi15g01552\_t001 |  | \| \| |  | \| \| |  |  |  |  |  |  |  |  |
| 2 | Vitvi15g00888\_t001 |  | \| \| |  | Vitvi02g00243\_t001 |  |  |  |  |  |  |  |  |
| 2 | Vitvi15g00889\_t001 |  | \| \| |  | Vitvi02g00242\_t001 |  |  |  |  |  |  |  |  |
| 2 | Vitvi15g00890\_t001 |  | \| \| |  | \| \| |  |  |  |  |  |  |  |  |
| 2 | Vitvi15g00893\_t001 |  | Vitvi16g01353\_t001 |  | Vitvi02g00240\_t001 |  |  |  |  |  |  |  |  |
| 2 | Vitvi15g00894\_t001 |  | \| \| |  | \| \| |  |  |  |  |  |  |  |  |
| 2 | Vitvi15g04476\_t001 |  | \| \| |  | \| \| |  |  |  |  |  |  |  |  |
| 2 | Vitvi15g00895\_t001 |  | \| \| |  | \| \| |  |  |  |  |  |  |  |  |
| 2 | Vitvi15g01553\_t001 |  | \| \| |  | \| \| |  |  |  |  |  |  |  |  |
| 2 | Vitvi15g00896\_t001 |  | \| \| |  | \| \| |  |  |  |  |  |  |  |  |
| 2 | Vitvi15g04477\_t001 |  | \| \| |  | \| \| |  |  |  |  |  |  |  |  |
| 2 | Vitvi15g00899\_t001 |  | \| \| |  | \| \| |  |  |  |  |  |  |  |  |
| 2 | Vitvi15g00900\_t001 |  | \| \| |  | \| \| |  |  |  |  |  |  |  |  |
| 2 | Vitvi15g04478\_t001 |  | \| \| |  | \| \| |  |  |  |  |  |  |  |  |
| 2 | Vitvi15g04479\_t001 |  | \| \| |  | \| \| |  |  |  |  |  |  |  |  |
| 2 | Vitvi15g00901\_t001 |  | \| \| |  | \| \| |  |  |  |  |  |  |  |  |
| 2 | Vitvi15g00902\_t001 |  | \| \| |  | \| \| |  |  |  |  |  |  |  |  |
| 2 | Vitvi15g00903\_t001 |  | Vitvi16g01357\_t001 |  | \| \| |  |  |  |  |  |  |  |  |
| 2 | Vitvi15g01555\_t001 |  | \| \| |  | \| \| |  |  |  |  |  |  |  |  |
| 2 | Vitvi15g04480\_t001 |  | \| \| |  | \| \| |  |  |  |  |  |  |  |  |
| 2 | Vitvi15g00905\_t001 |  | \| \| |  | \| \| |  |  |  |  |  |  |  |  |
| 2 | Vitvi15g00906\_t001 |  | \| \| |  | Vitvi02g00231\_t001 |  |  |  |  |  |  |  |  |
| 2 | Vitvi15g04481\_t001 |  | \| \| |  | \| \| |  |  |  |  |  |  |  |  |
| 2 | Vitvi15g00907\_t001 |  | \| \| |  | \| \| |  |  |  |  |  |  |  |  |
| 2 | Vitvi15g00908\_t001 |  | Vitvi16g01358\_t001 |  | \| \| |  |  |  |  |  |  |  |  |
| 2 | Vitvi15g00909\_t001 |  | \| \| |  | \| \| |  |  |  |  |  |  |  |  |
| 2 | Vitvi15g00910\_t001 |  | Vitvi16g01359\_t001 |  | \| \| |  |  |  |  |  |  |  |  |
| 2 | Vitvi15g04482\_t001 |  | \| \| |  | \| \| |  |  |  |  |  |  |  |  |
| 2 | Vitvi15g00912\_t001 |  | Vitvi16g01362\_t001 |  | Vitvi02g00228\_t001 |  |  |  |  |  |  |  |  |
| 2 | Vitvi15g00913\_t001 |  | \| \| |  | \| \| |  |  |  |  |  |  |  |  |
| 2 | Vitvi15g00915\_t001 |  | \| \| |  | Vitvi02g00226\_t001 |  |  |  |  |  |  |  |  |
| 1 | Vitvi15g00916\_t001 |  | \| \| |  |  |  |  |  |  |  |  |  |  |
| 1 | Vitvi15g01559\_t001 |  | \| \| |  |  |  |  |  |  |  |  |  |  |
| 1 | Vitvi15g01560\_t001 |  | \| \| |  |  |  |  |  |  |  |  |  |  |
| 1 | Vitvi15g01561\_t001 |  | \| \| |  |  |  |  |  |  |  |  |  |  |
| 1 | Vitvi15g01562\_t001 |  | \| \| |  |  |  |  |  |  |  |  |  |  |
| 1 | Vitvi15g04483\_t001 |  | \| \| |  |  |  |  |  |  |  |  |  |  |
| 1 | Vitvi15g01564\_t001 |  | \| \| |  |  |  |  |  |  |  |  |  |  |
| 1 | Vitvi15g04484\_t001 |  | \| \| |  |  |  |  |  |  |  |  |  |  |
| 1 | Vitvi15g04485\_t001 |  | \| \| |  |  |  |  |  |  |  |  |  |  |
| 1 | Vitvi15g04486\_t001 |  | \| \| |  |  |  |  |  |  |  |  |  |  |
| 1 | Vitvi15g01565\_t001 |  | \| \| |  |  |  |  |  |  |  |  |  |  |
| 1 | Vitvi15g00919\_t001 |  | \| \| |  |  |  |  |  |  |  |  |  |  |
| 1 | Vitvi15g00920\_t001 |  | Vitvi16g01374\_t001 |  |  |  |  |  |  |  |  |  |  |
| 0 | Vitvi15g00921\_t001 |  |  |  |  |  |  |  |  |  |  |  |  |
| 0 | Vitvi15g01566\_t001 |  |  |  |  |  |  |  |  |  |  |  |  |
| 0 | Vitvi15g04487\_t001 |  |  |  |  |  |  |  |  |  |  |  |  |
| 0 | Vitvi15g04488\_t001 |  |  |  |  |  |  |  |  |  |  |  |  |
| 0 | Vitvi15g04489\_t001 |  |  |  |  |  |  |  |  |  |  |  |  |
| 0 | Vitvi15g04490\_t001 |  |  |  |  |  |  |  |  |  |  |  |  |
| 0 | Vitvi15g04491\_t001 |  |  |  |  |  |  |  |  |  |  |  |  |
| 0 | Vitvi15g04492\_t001 |  |  |  |  |  |  |  |  |  |  |  |  |
| 0 | Vitvi15g04493\_t001 |  |  |  |  |  |  |  |  |  |  |  |  |
| 1 | Vitvi15g04494\_t001 |  | Vitvi18g00645\_t003 |  |  |  |  |  |  |  |  |  |  |
| 1 | Vitvi15g04495\_t001 |  | \| \| |  |  |  |  |  |  |  |  |  |  |
| 1 | Vitvi15g04496\_t001 |  | \| \| |  |  |  |  |  |  |  |  |  |  |
| 1 | Vitvi15g04497\_t001 |  | \| \| |  |  |  |  |  |  |  |  |  |  |
| 1 | Vitvi15g04498\_t001 |  | \| \| |  |  |  |  |  |  |  |  |  |  |
| 1 | Vitvi15g04499\_t001 |  | \| \| |  |  |  |  |  |  |  |  |  |  |
| 1 | Vitvi15g04500\_t001 |  | \| \| |  |  |  |  |  |  |  |  |  |  |
| 1 | Vitvi15g04501\_t001 |  | Vitvi18g02636\_t001 |  |  |  |  |  |  |  |  |  |  |
| 1 | Vitvi15g04502\_t001 |  | \| \| |  |  |  |  |  |  |  |  |  |  |
| 1 | Vitvi15g04503\_t001 |  | Vitvi18g00638\_t001 |  |  |  |  |  |  |  |  |  |  |
| 1 | Vitvi15g04504\_t001 |  | Vitvi18g02635\_t001 |  |  |  |  |  |  |  |  |  |  |
| 1 | Vitvi15g04505\_t001 |  | \| \| |  |  |  |  |  |  |  |  |  |  |
| 1 | Vitvi15g04506\_t001 |  | Vitvi18g00637\_t001 |  |  |  |  |  |  |  |  |  |  |
| 1 | Vitvi15g04507\_t001 |  | \| \| |  |  |  |  |  |  |  |  |  |  |
| 1 | Vitvi15g04508\_t001 |  | Vitvi18g00636\_t001 |  |  |  |  |  |  |  |  |  |  |
| 1 | Vitvi15g04509\_t001 |  | \| \| |  |  |  |  |  |  |  |  |  |  |
| 1 | Vitvi15g04510\_t001 |  | \| \| |  |  |  |  |  |  |  |  |  |  |
| 1 | Vitvi15g04511\_t001 |  | Vitvi18g00635\_t001 |  |  |  |  |  |  |  |  |  |  |
| 1 | Vitvi15g04512\_t001 |  | Vitvi18g00634\_t001 |  |  |  |  |  |  |  |  |  |  |
| 1 | Vitvi15g04513\_t001 |  | \| \| |  |  |  |  |  |  |  |  |  |  |
| 1 | Vitvi15g04514\_t001 |  | Vitvi18g00628\_t001 |  |  |  |  |  |  |  |  |  |  |
| 1 | Vitvi15g04515\_t001 |  | Vitvi18g02631\_t001 |  |  |  |  |  |  |  |  |  |  |
| 1 | Vitvi15g04516\_t001 |  | \| \| |  |  |  |  |  |  |  |  |  |  |
| 1 | Vitvi15g04517\_t001 |  | Vitvi18g00623\_t001 |  |  |  |  |  |  |  |  |  |  |
| 1 | Vitvi15g04518\_t001 |  | \| \| |  |  |  |  |  |  |  |  |  |  |
| 1 | Vitvi15g04519\_t003 |  | \| \| |  |  |  |  |  |  |  |  |  |  |
| 1 | Vitvi15g04520\_t001 |  | \| \| |  |  |  |  |  |  |  |  |  |  |
| 1 | Vitvi15g04521\_t001 |  | Vitvi18g00618\_t001 |  |  |  |  |  |  |  |  |  |  |
| 1 | Vitvi15g04522\_t001 |  | Vitvi18g00617\_t001 |  |  |  |  |  |  |  |  |  |  |
| 1 | Vitvi15g04523\_t001 |  | \| \| |  |  |  |  |  |  |  |  |  |  |
| 1 | Vitvi15g04524\_t001 |  | \| \| |  |  |  |  |  |  |  |  |  |  |
| 1 | Vitvi15g04525\_t001 |  | \| \| |  |  |  |  |  |  |  |  |  |  |
| 1 | Vitvi15g04526\_t001 |  | Vitvi18g00612\_t001 |  |  |  |  |  |  |  |  |  |  |
| 1 | Vitvi15g04527\_t001 |  | \| \| |  |  |  |  |  |  |  |  |  |  |
| 1 | Vitvi15g04528\_t001 |  | \| \| |  |  |  |  |  |  |  |  |  |  |
| 1 | Vitvi15g04529\_t001 |  | \| \| |  |  |  |  |  |  |  |  |  |  |
| 1 | Vitvi15g04530\_t001 |  | \| \| |  |  |  |  |  |  |  |  |  |  |
| 1 | Vitvi15g04531\_t001 |  | \| \| |  |  |  |  |  |  |  |  |  |  |
| 1 | Vitvi15g04532\_t001 |  | \| \| |  |  |  |  |  |  |  |  |  |  |
| 1 | Vitvi15g04533\_t001 |  | \| \| |  |  |  |  |  |  |  |  |  |  |
| 1 | Vitvi15g04534\_t001 |  | \| \| |  |  |  |  |  |  |  |  |  |  |
| 1 | Vitvi15g04535\_t001 |  | Vitvi18g00610\_t001 |  |  |  |  |  |  |  |  |  |  |
| 0 | Vitvi15g04536\_t001 |  |  |  |  |  |  |  |  |  |  |  |  |
| 0 | Vitvi15g04537\_t001 |  |  |  |  |  |  |  |  |  |  |  |  |
| 0 | Vitvi15g04538\_t001 |  |  |  |  |  |  |  |  |  |  |  |  |
| 0 | Vitvi15g04539\_t001 |  |  |  |  |  |  |  |  |  |  |  |  |
| 0 | Vitvi15g01758\_t001 |  |  |  |  |  |  |  |  |  |  |  |  |
| 0 | Vitvi15g04540\_t001 |  |  |  |  |  |  |  |  |  |  |  |  |
| 0 | Vitvi15g04541\_t001 |  |  |  |  |  |  |  |  |  |  |  |  |
| 0 | Vitvi15g01762\_t001 |  |  |  |  |  |  |  |  |  |  |  |  |
| 0 | Vitvi15g04542\_t001 |  |  |  |  |  |  |  |  |  |  |  |  |
| 0 | Vitvi15g04543\_t001 |  |  |  |  |  |  |  |  |  |  |  |  |
| 0 | Vitvi15g04544\_t001 |  |  |  |  |  |  |  |  |  |  |  |  |
| 0 | Vitvi15g04545\_t001 |  |  |  |  |  |  |  |  |  |  |  |  |
| 0 | Vitvi15g04546\_t001 |  |  |  |  |  |  |  |  |  |  |  |  |
| 0 | Vitvi15g04547\_t001 |  |  |  |  |  |  |  |  |  |  |  |  |
| 0 | Vitvi15g01567\_t001 |  |  |  |  |  |  |  |  |  |  |  |  |
| 0 | Vitvi15g04548\_t001 |  |  |  |  |  |  |  |  |  |  |  |  |
| 1 | Vitvi15g00923\_t001 |  | Vitvi16g01379\_t001 |  |  |  |  |  |  |  |  |  |  |
| 1 | Vitvi15g00924\_t001 |  | \| \| |  |  |  |  |  |  |  |  |  |  |
| 1 | Vitvi15g00925\_t001 |  | \| \| |  |  |  |  |  |  |  |  |  |  |
| 1 | Vitvi15g00926\_t003 |  | \| \| |  |  |  |  |  |  |  |  |  |  |
| 1 | Vitvi15g00928\_t001 |  | \| \| |  |  |  |  |  |  |  |  |  |  |
| 1 | Vitvi15g04549\_t001 |  | \| \| |  |  |  |  |  |  |  |  |  |  |
| 2 | Vitvi15g00929\_t001 |  | Vitvi16g04515\_t001 |  | Vitvi02g00208\_t002 |  |  |  |  |  |  |  |  |
| 2 | Vitvi15g00930\_t002 |  | \| \| |  | \| \| |  |  |  |  |  |  |  |  |
| 2 | Vitvi15g00931\_t002 |  | \| \| |  | Vitvi02g00207\_t001 |  |  |  |  |  |  |  |  |
| 2 | Vitvi15g00932\_t001 |  | \| \| |  | \| \| |  |  |  |  |  |  |  |  |
| 2 | Vitvi15g00933\_t001 |  | \| \| |  | \| \| |  |  |  |  |  |  |  |  |
| 2 | Vitvi15g00934\_t001 |  | \| \| |  | Vitvi02g00203\_t001 |  |  |  |  |  |  |  |  |
| 2 | Vitvi15g00935\_t001 |  | \| \| |  | Vitvi02g00200\_t001 |  |  |  |  |  |  |  |  |
| 2 | Vitvi15g00936\_t001 |  | Vitvi16g01384\_t001 |  | Vitvi02g00199\_t001 |  |  |  |  |  |  |  |  |
| 2 | Vitvi15g00937\_t001 |  | Vitvi16g01387\_t001 |  | Vitvi02g00198\_t001 |  |  |  |  |  |  |  |  |
| 2 | Vitvi15g00938\_t001 |  | \| \| |  | Vitvi02g00195\_t001 |  |  |  |  |  |  |  |  |
| 2 | Vitvi15g00940\_t001 |  | \| \| |  | \| \| |  |  |  |  |  |  |  |  |
| 2 | Vitvi15g00941\_t001 |  | \| \| |  | \| \| |  |  |  |  |  |  |  |  |
| 2 | Vitvi15g04550\_t001 |  | Vitvi16g02080\_t001 |  | \| \| |  |  |  |  |  |  |  |  |
| 2 | Vitvi15g04551\_t001 |  | \| \| |  | \| \| |  |  |  |  |  |  |  |  |
| 2 | Vitvi15g00942\_t001 |  | \| \| |  | \| \| |  |  |  |  |  |  |  |  |
| 2 | Vitvi15g04552\_t001 |  | \| \| |  | \| \| |  |  |  |  |  |  |  |  |
| 2 | Vitvi15g00943\_t001 |  | \| \| |  | Vitvi02g00182\_t001 |  |  |  |  |  |  |  |  |
| 1 | Vitvi15g04553\_t001 |  | \| \| |  |  |  |  |  |  |  |  |  |  |
| 1 | Vitvi15g04554\_t001 |  | \| \| |  |  |  |  |  |  |  |  |  |  |
| 1 | Vitvi15g01217\_t001 |  | \| \| |  |  |  |  |  |  |  |  |  |  |
| 1 | Vitvi15g00944\_t001 |  | \| \| |  |  |  |  |  |  |  |  |  |  |
| 1 | Vitvi15g00945\_t008 |  | Vitvi16g02085\_t001 |  |  |  |  |  |  |  |  |  |  |
| 1 | Vitvi15g01569\_t001 |  | \| \| |  |  |  |  |  |  |  |  |  |  |
| 1 | Vitvi15g01570\_t001 |  | Vitvi16g02087\_t001 |  |  |  |  |  |  |  |  |  |  |
| 1 | Vitvi15g04555\_t001 |  | \| \| |  |  |  |  |  |  |  |  |  |  |
| 1 | Vitvi15g00946\_t001 |  | \| \| |  |  |  |  |  |  |  |  |  |  |
| 1 | Vitvi15g01571\_t002 |  | \| \| |  |  |  |  |  |  |  |  |  |  |
| 1 | Vitvi15g00947\_t001 |  | \| \| |  |  |  |  |  |  |  |  |  |  |
| 1 | Vitvi15g00948\_t001 |  | \| \| |  |  |  |  |  |  |  |  |  |  |
| 1 | Vitvi15g00949\_t001 |  | \| \| |  |  |  |  |  |  |  |  |  |  |
| 1 | Vitvi15g00950\_t001 |  | Vitvi16g01410\_t001 |  |  |  |  |  |  |  |  |  |  |
| 1 | Vitvi15g00951\_t001 |  | \| \| |  |  |  |  |  |  |  |  |  |  |
| 1 | Vitvi15g00952\_t001 |  | \| \| |  |  |  |  |  |  |  |  |  |  |
| 1 | Vitvi15g00953\_t001 |  | \| \| |  |  |  |  |  |  |  |  |  |  |
| 1 | Vitvi15g04556\_t001 |  | \| \| |  |  |  |  |  |  |  |  |  |  |
| 1 | Vitvi15g04557\_t001 |  | \| \| |  |  |  |  |  |  |  |  |  |  |
| 1 | Vitvi15g01573\_t001 |  | \| \| |  |  |  |  |  |  |  |  |  |  |
| 1 | Vitvi15g01574\_t001 |  | Vitvi16g01412\_t001 |  |  |  |  |  |  |  |  |  |  |
| 1 | Vitvi15g04558\_t001 |  | \| \| |  |  |  |  |  |  |  |  |  |  |
| 1 | Vitvi15g00954\_t002 |  | \| \| |  |  |  |  |  |  |  |  |  |  |
| 1 | Vitvi15g00955\_t001 |  | \| \| |  |  |  |  |  |  |  |  |  |  |
| 1 | Vitvi15g01576\_t001 |  | Vitvi16g01417\_t003 |  |  |  |  |  |  |  |  |  |  |
| 0 | Vitvi15g00956\_t001 |  |  |  |  |  |  |  |  |  |  |  |  |
| 0 | Vitvi15g00957\_t001 |  |  |  |  |  |  |  |  |  |  |  |  |
| 0 | Vitvi15g00958\_t001 |  |  |  |  |  |  |  |  |  |  |  |  |
| 0 | Vitvi15g04559\_t001 |  |  |  |  |  |  |  |  |  |  |  |  |
| 0 | Vitvi15g00960\_t002 |  |  |  |  |  |  |  |  |  |  |  |  |
| 0 | Vitvi15g04560\_t001 |  |  |  |  |  |  |  |  |  |  |  |  |
| 0 | Vitvi15g00968\_t001 |  |  |  |  |  |  |  |  |  |  |  |  |
| 0 | Vitvi15g00969\_t001 |  |  |  |  |  |  |  |  |  |  |  |  |
| 0 | Vitvi15g04561\_t001 |  |  |  |  |  |  |  |  |  |  |  |  |
| 0 | Vitvi15g04562\_t001 |  |  |  |  |  |  |  |  |  |  |  |  |
| 0 | Vitvi15g01580\_t001 |  |  |  |  |  |  |  |  |  |  |  |  |
| 0 | Vitvi15g01582\_t001 |  |  |  |  |  |  |  |  |  |  |  |  |
| 0 | Vitvi15g01583\_t001 |  |  |  |  |  |  |  |  |  |  |  |  |
| 0 | Vitvi15g01584\_t001 |  |  |  |  |  |  |  |  |  |  |  |  |
| 0 | Vitvi15g04563\_t001 |  |  |  |  |  |  |  |  |  |  |  |  |
| 0 | Vitvi15g04565\_t001 |  |  |  |  |  |  |  |  |  |  |  |  |
| 0 | Vitvi15g04564\_t001 |  |  |  |  |  |  |  |  |  |  |  |  |
| 0 | Vitvi15g00972\_t001 |  |  |  |  |  |  |  |  |  |  |  |  |
| 0 | Vitvi15g00973\_t001 |  |  |  |  |  |  |  |  |  |  |  |  |
| 0 | Vitvi15g04566\_t001 |  |  |  |  |  |  |  |  |  |  |  |  |
| 0 | Vitvi15g00974\_t001 |  |  |  |  |  |  |  |  |  |  |  |  |
| 0 | Vitvi15g00975\_t001 |  |  |  |  |  |  |  |  |  |  |  |  |
| 0 | Vitvi15g04567\_t001 |  |  |  |  |  |  |  |  |  |  |  |  |
| 0 | Vitvi15g00976\_t001 |  |  |  |  |  |  |  |  |  |  |  |  |
| 0 | Vitvi15g01588\_t001 |  |  |  |  |  |  |  |  |  |  |  |  |
| 0 | Vitvi15g01589\_t001 |  |  |  |  |  |  |  |  |  |  |  |  |
| 0 | Vitvi15g04568\_t001 |  |  |  |  |  |  |  |  |  |  |  |  |
| 0 | Vitvi15g04569\_t001 |  |  |  |  |  |  |  |  |  |  |  |  |
| 0 | Vitvi15g01590\_t001 |  |  |  |  |  |  |  |  |  |  |  |  |
| 0 | Vitvi15g04570\_t001 |  |  |  |  |  |  |  |  |  |  |  |  |
| 0 | Vitvi15g01591\_t001 |  |  |  |  |  |  |  |  |  |  |  |  |
| 0 | Vitvi15g01592\_t001 |  |  |  |  |  |  |  |  |  |  |  |  |
| 0 | Vitvi15g04571\_t001 |  |  |  |  |  |  |  |  |  |  |  |  |
| 0 | Vitvi15g01593\_t001 |  |  |  |  |  |  |  |  |  |  |  |  |
| 0 | Vitvi15g04572\_t001 |  |  |  |  |  |  |  |  |  |  |  |  |
| 0 | Vitvi15g01595\_t001 |  |  |  |  |  |  |  |  |  |  |  |  |
| 0 | Vitvi15g04573\_t001 |  |  |  |  |  |  |  |  |  |  |  |  |
| 0 | Vitvi15g04574\_t001 |  |  |  |  |  |  |  |  |  |  |  |  |
| 0 | Vitvi15g04575\_t001 |  |  |  |  |  |  |  |  |  |  |  |  |
| 0 | Vitvi15g04576\_t001 |  |  |  |  |  |  |  |  |  |  |  |  |
| 0 | Vitvi15g01598\_t001 |  |  |  |  |  |  |  |  |  |  |  |  |
| 0 | Vitvi15g04577\_t001 |  |  |  |  |  |  |  |  |  |  |  |  |
| 0 | Vitvi15g04578\_t001 |  |  |  |  |  |  |  |  |  |  |  |  |
| 0 | Vitvi15g04579\_t001 |  |  |  |  |  |  |  |  |  |  |  |  |
| 0 | Vitvi15g00981\_t001 |  |  |  |  |  |  |  |  |  |  |  |  |
| 0 | Vitvi15g04580\_t001 |  |  |  |  |  |  |  |  |  |  |  |  |
| 0 | Vitvi15g04581\_t001 |  |  |  |  |  |  |  |  |  |  |  |  |
| 0 | Vitvi15g04582\_t001 |  |  |  |  |  |  |  |  |  |  |  |  |
| 0 | Vitvi15g01602\_t001 |  |  |  |  |  |  |  |  |  |  |  |  |
| 0 | Vitvi15g04583\_t001 |  |  |  |  |  |  |  |  |  |  |  |  |
| 0 | Vitvi15g04584\_t001 |  |  |  |  |  |  |  |  |  |  |  |  |
| 0 | Vitvi15g01604\_t001 |  |  |  |  |  |  |  |  |  |  |  |  |
| 0 | Vitvi15g04585\_t001 |  |  |  |  |  |  |  |  |  |  |  |  |
| 0 | Vitvi15g01605\_t001 |  |  |  |  |  |  |  |  |  |  |  |  |
| 0 | Vitvi15g01606\_t001 |  |  |  |  |  |  |  |  |  |  |  |  |
| 0 | Vitvi15g01607\_t001 |  |  |  |  |  |  |  |  |  |  |  |  |
| 0 | Vitvi15g01608\_t001 |  |  |  |  |  |  |  |  |  |  |  |  |
| 0 | Vitvi15g01609\_t001 |  |  |  |  |  |  |  |  |  |  |  |  |
| 0 | Vitvi15g01610\_t001 |  |  |  |  |  |  |  |  |  |  |  |  |
| 0 | Vitvi15g01611\_t001 |  |  |  |  |  |  |  |  |  |  |  |  |
| 0 | Vitvi15g04586\_t001 |  |  |  |  |  |  |  |  |  |  |  |  |
| 0 | Vitvi15g04587\_t001 |  |  |  |  |  |  |  |  |  |  |  |  |
| 0 | Vitvi15g04588\_t001 |  |  |  |  |  |  |  |  |  |  |  |  |
| 0 | Vitvi15g04589\_t001 |  |  |  |  |  |  |  |  |  |  |  |  |
| 1 | Vitvi15g01614\_t001 |  | Vitvi02g00130\_t001 |  |  |  |  |  |  |  |  |  |  |
| 1 | Vitvi15g00985\_t001 |  | \| \| |  |  |  |  |  |  |  |  |  |  |
| 1 | Vitvi15g00986\_t001 |  | \| \| |  |  |  |  |  |  |  |  |  |  |
| 1 | Vitvi15g00987\_t001 |  | \| \| |  |  |  |  |  |  |  |  |  |  |
| 1 | Vitvi15g01615\_t001 |  | \| \| |  |  |  |  |  |  |  |  |  |  |
| 1 | Vitvi15g01616\_t001 |  | \| \| |  |  |  |  |  |  |  |  |  |  |
| 1 | Vitvi15g00989\_t001 |  | \| \| |  |  |  |  |  |  |  |  |  |  |
| 1 | Vitvi15g01617\_t001 |  | \| \| |  |  |  |  |  |  |  |  |  |  |
| 1 | Vitvi15g00990\_t001 |  | \| \| |  |  |  |  |  |  |  |  |  |  |
| 1 | Vitvi15g04590\_t001 |  | \| \| |  |  |  |  |  |  |  |  |  |  |
| 1 | Vitvi15g00991\_t001 |  | \| \| |  |  |  |  |  |  |  |  |  |  |
| 1 | Vitvi15g00992\_t001 |  | \| \| |  |  |  |  |  |  |  |  |  |  |
| 1 | Vitvi15g00993\_t001 |  | \| \| |  |  |  |  |  |  |  |  |  |  |
| 1 | Vitvi15g01618\_t001 |  | \| \| |  |  |  |  |  |  |  |  |  |  |
| 1 | Vitvi15g00994\_t001 |  | \| \| |  |  |  |  |  |  |  |  |  |  |
| 1 | Vitvi15g00995\_t001 |  | \| \| |  |  |  |  |  |  |  |  |  |  |
| 1 | Vitvi15g04591\_t001 |  | \| \| |  |  |  |  |  |  |  |  |  |  |
| 1 | Vitvi15g04592\_t001 |  | \| \| |  |  |  |  |  |  |  |  |  |  |
| 1 | Vitvi15g00997\_t001 |  | Vitvi02g00119\_t001 |  |  |  |  |  |  |  |  |  |  |
| 1 | Vitvi15g00998\_t001 |  | \| \| |  |  |  |  |  |  |  |  |  |  |
| 1 | Vitvi15g00999\_t001 |  | \| \| |  |  |  |  |  |  |  |  |  |  |
| 1 | Vitvi15g01000\_t002 |  | \| \| |  |  |  |  |  |  |  |  |  |  |
| 1 | Vitvi15g01619\_t001 |  | \| \| |  |  |  |  |  |  |  |  |  |  |
| 1 | Vitvi15g01621\_t001 |  | \| \| |  |  |  |  |  |  |  |  |  |  |
| 1 | Vitvi15g01001\_t001 |  | \| \| |  |  |  |  |  |  |  |  |  |  |
| 1 | Vitvi15g01002\_t001 |  | \| \| |  |  |  |  |  |  |  |  |  |  |
| 1 | Vitvi15g01003\_t001 |  | Vitvi02g00114\_t001 |  |  |  |  |  |  |  |  |  |  |
| 1 | Vitvi15g01005\_t001 |  | Vitvi02g00112\_t001 |  |  |  |  |  |  |  |  |  |  |
| 1 | Vitvi15g01006\_t001 |  | \| \| |  |  |  |  |  |  |  |  |  |  |
| 1 | Vitvi15g01007\_t001 |  | \| \| |  |  |  |  |  |  |  |  |  |  |
| 1 | Vitvi15g01623\_t001 |  | Vitvi02g00110\_t001 |  |  |  |  |  |  |  |  |  |  |
| 1 | Vitvi15g01624\_t001 |  | \| \| |  |  |  |  |  |  |  |  |  |  |
| 1 | Vitvi15g01008\_t001 |  | \| \| |  |  |  |  |  |  |  |  |  |  |
| 1 | Vitvi15g04593\_t001 |  | \| \| |  |  |  |  |  |  |  |  |  |  |
| 1 | Vitvi15g01011\_t001 |  | Vitvi02g00106\_t001 |  |  |  |  |  |  |  |  |  |  |
| 1 | Vitvi15g01013\_t001 |  | Vitvi02g00105\_t001 |  |  |  |  |  |  |  |  |  |  |
| 1 | Vitvi15g01626\_t001 |  | Vitvi02g04016\_t001 |  |  |  |  |  |  |  |  |  |  |
| 1 | Vitvi15g01014\_t003 |  | \| \| |  |  |  |  |  |  |  |  |  |  |
| 1 | Vitvi15g01015\_t001 |  | Vitvi02g00103\_t001 |  |  |  |  |  |  |  |  |  |  |
| 1 | Vitvi15g01627\_t001 |  | \| \| |  |  |  |  |  |  |  |  |  |  |
| 1 | Vitvi15g04594\_t001 |  | \| \| |  |  |  |  |  |  |  |  |  |  |
| 1 | Vitvi15g01016\_t002 |  | Vitvi02g00098\_t001 |  |  |  |  |  |  |  |  |  |  |
| 1 | Vitvi15g01628\_t001 |  | Vitvi02g00097\_t001 |  |  |  |  |  |  |  |  |  |  |
| 1 | Vitvi15g01017\_t001 |  | \| \| |  |  |  |  |  |  |  |  |  |  |
| 2 | Vitvi15g01629\_t001 |  | Vitvi02g00096\_t001 |  | Vitvi16g01982\_t001 |  |  |  |  |  |  |  |  |
| 2 | Vitvi15g01018\_t001 |  | \| \| |  | \| \| |  |  |  |  |  |  |  |  |
| 2 | Vitvi15g01019\_t001 |  | Vitvi02g01335\_t001 |  | Vitvi16g01191\_t001 |  |  |  |  |  |  |  |  |
| 2 | Vitvi15g01020\_t001 |  | \| \| |  | Vitvi16g01197\_t001 |  |  |  |  |  |  |  |  |
| 2 | Vitvi15g01630\_t001 |  | \| \| |  | Vitvi16g01983\_t001 |  |  |  |  |  |  |  |  |
| 2 | Vitvi15g01021\_t001 |  | Vitvi02g00093\_t001 |  | Vitvi16g01201\_t001 |  |  |  |  |  |  |  |  |
| 2 | Vitvi15g04595\_t001 |  | Vitvi02g04013\_t001 |  | Vitvi16g04430\_t001 |  |  |  |  |  |  |  |  |
| 1 | Vitvi15g01024\_t001 |  | \| \| |  |  |  |  |  |  |  |  |  |  |
| 1 | Vitvi15g04596\_t001 |  | \| \| |  |  |  |  |  |  |  |  |  |  |
| 1 | Vitvi15g01025\_t001 |  | \| \| |  |  |  |  |  |  |  |  |  |  |
| 1 | Vitvi15g01026\_t001 |  | Vitvi02g00090\_t001 |  |  |  |  |  |  |  |  |  |  |
| 1 | Vitvi15g01027\_t001 |  | Vitvi02g00089\_t001 |  |  |  |  |  |  |  |  |  |  |
| 1 | Vitvi15g01028\_t001 |  | \| \| |  |  |  |  |  |  |  |  |  |  |
| 1 | Vitvi15g01029\_t001 |  | Vitvi02g00079\_t001 |  |  |  |  |  |  |  |  |  |  |
| 1 | Vitvi15g01030\_t001 |  | \| \| |  |  |  |  |  |  |  |  |  |  |
| 1 | Vitvi15g01631\_t001 |  | \| \| |  |  |  |  |  |  |  |  |  |  |
| 1 | Vitvi15g04597\_t001 |  | \| \| |  |  |  |  |  |  |  |  |  |  |
| 1 | Vitvi15g01031\_t001 |  | Vitvi02g00078\_t001 |  |  |  |  |  |  |  |  |  |  |
| 1 | Vitvi15g01032\_t001 |  | Vitvi02g01329\_t001 |  |  |  |  |  |  |  |  |  |  |
| 1 | Vitvi15g01033\_t003 |  | Vitvi02g00075\_t001 |  |  |  |  |  |  |  |  |  |  |
| 1 | Vitvi15g01634\_t001 |  | \| \| |  |  |  |  |  |  |  |  |  |  |
| 1 | Vitvi15g01034\_t002 |  | \| \| |  |  |  |  |  |  |  |  |  |  |
| 1 | Vitvi15g01037\_t001 |  | \| \| |  |  |  |  |  |  |  |  |  |  |
| 1 | Vitvi15g04598\_t001 |  | \| \| |  |  |  |  |  |  |  |  |  |  |
| 1 | Vitvi15g04599\_t001 |  | \| \| |  |  |  |  |  |  |  |  |  |  |
| 1 | Vitvi15g01045\_t001 |  | \| \| |  |  |  |  |  |  |  |  |  |  |
| 1 | Vitvi15g01046\_t001 |  | \| \| |  |  |  |  |  |  |  |  |  |  |
| 1 | Vitvi15g01047\_t001 |  | \| \| |  |  |  |  |  |  |  |  |  |  |
| 1 | Vitvi15g04600\_t001 |  | \| \| |  |  |  |  |  |  |  |  |  |  |
| 1 | Vitvi15g01048\_t001 |  | \| \| |  |  |  |  |  |  |  |  |  |  |
| 1 | Vitvi15g01636\_t001 |  | \| \| |  |  |  |  |  |  |  |  |  |  |
| 1 | Vitvi15g01049\_t001 |  | \| \| |  |  |  |  |  |  |  |  |  |  |
| 1 | Vitvi15g01050\_t001 |  | \| \| |  |  |  |  |  |  |  |  |  |  |
| 1 | Vitvi15g01637\_t001 |  | \| \| |  |  |  |  |  |  |  |  |  |  |
| 1 | Vitvi15g01051\_t001 |  | \| \| |  |  |  |  |  |  |  |  |  |  |
| 1 | Vitvi15g01638\_t001 |  | \| \| |  |  |  |  |  |  |  |  |  |  |
| 1 | Vitvi15g01639\_t001 |  | \| \| |  |  |  |  |  |  |  |  |  |  |
| 1 | Vitvi15g01052\_t001 |  | \| \| |  |  |  |  |  |  |  |  |  |  |
| 1 | Vitvi15g01053\_t001 |  | \| \| |  |  |  |  |  |  |  |  |  |  |
| 1 | Vitvi15g01640\_t001.1.6037826c |  | Vitvi02g00059\_t001 |  |  |  |  |  |  |  |  |  |  |
| 0 | Vitvi15g01054\_t002 |  |  |  |  |  |  |  |  |  |  |  |  |
| 0 | Vitvi15g01055\_t001 |  |  |  |  |  |  |  |  |  |  |  |  |
| 0 | Vitvi15g01056\_t001 |  |  |  |  |  |  |  |  |  |  |  |  |
| 1 | Vitvi15g01057\_t001 |  | Vitvi16g01911\_t001 |  |  |  |  |  |  |  |  |  |  |
| 1 | Vitvi15g01058\_t001 |  | \| \| |  |  |  |  |  |  |  |  |  |  |
| 1 | Vitvi15g04601\_t001 |  | \| \| |  |  |  |  |  |  |  |  |  |  |
| 2 | Vitvi15g01059\_t001 |  | \| \| |  | Vitvi02g00001\_t001 |  |  |  |  |  |  |  |  |
| 2 | Vitvi15g01061\_t001 |  | \| \| |  | \| \| |  |  |  |  |  |  |  |  |
| 2 | Vitvi15g01060\_t001 |  | \| \| |  | \| \| |  |  |  |  |  |  |  |  |
| 2 | Vitvi15g01062\_t001 |  | \| \| |  | \| \| |  |  |  |  |  |  |  |  |
| 2 | Vitvi15g01063\_t001 |  | \| \| |  | \| \| |  |  |  |  |  |  |  |  |
| 2 | Vitvi15g01065\_t001 |  | \| \| |  | \| \| |  |  |  |  |  |  |  |  |
| 2 | Vitvi15g01066\_t001 |  | \| \| |  | Vitvi02g04003\_t002 |  |  |  |  |  |  |  |  |
| 2 | Vitvi15g01067\_t001 |  | \| \| |  | Vitvi02g00013\_t001 |  |  |  |  |  |  |  |  |
| 2 | Vitvi15g01068\_t001 |  | \| \| |  | \| \| |  |  |  |  |  |  |  |  |
| 2 | Vitvi15g01069\_t003 |  | Vitvi16g01090\_t001 |  | \| \| |  |  |  |  |  |  |  |  |
| 2 | Vitvi15g04602\_t001 |  | \| \| |  | \| \| |  |  |  |  |  |  |  |  |
| 2 | Vitvi15g01070\_t001 |  | \| \| |  | \| \| |  |  |  |  |  |  |  |  |
| 2 | Vitvi15g01071\_t001 |  | Vitvi16g01094\_t001 |  | \| \| |  |  |  |  |  |  |  |  |
| 2 | Vitvi15g04603\_t001 |  | \| \| |  | \| \| |  |  |  |  |  |  |  |  |
| 2 | Vitvi15g01072\_t001 |  | \| \| |  | \| \| |  |  |  |  |  |  |  |  |
| 2 | Vitvi15g01641\_t001 |  | \| \| |  | \| \| |  |  |  |  |  |  |  |  |
| 2 | Vitvi15g01642\_t001 |  | \| \| |  | \| \| |  |  |  |  |  |  |  |  |
| 2 | Vitvi15g01073\_t001 |  | \| \| |  | \| \| |  |  |  |  |  |  |  |  |
| 2 | Vitvi15g04604\_t001 |  | \| \| |  | \| \| |  |  |  |  |  |  |  |  |
| 2 | Vitvi15g01075\_t001 |  | \| \| |  | \| \| |  |  |  |  |  |  |  |  |
| 2 | Vitvi15g04605\_t001 |  | \| \| |  | \| \| |  |  |  |  |  |  |  |  |
| 2 | Vitvi15g04606\_t001 |  | \| \| |  | \| \| |  |  |  |  |  |  |  |  |
| 2 | Vitvi15g04607\_t001 |  | Vitvi16g01921\_t001 |  | Vitvi02g00022\_t001 |  |  |  |  |  |  |  |  |
| 2 | Vitvi15g01077\_t001 |  | \| \| |  | Vitvi02g00023\_t001 |  |  |  |  |  |  |  |  |
| 2 | Vitvi15g01644\_t003 |  | \| \| |  | \| \| |  |  |  |  |  |  |  |  |
| 2 | Vitvi15g01078\_t001 |  | Vitvi16g01101\_t001 |  | \| \| |  |  |  |  |  |  |  |  |
| 2 | Vitvi15g04608\_t001 |  | \| \| |  | \| \| |  |  |  |  |  |  |  |  |
| 2 | Vitvi15g01080\_t001 |  | \| \| |  | \| \| |  |  |  |  |  |  |  |  |
| 2 | Vitvi15g01081\_t001 |  | \| \| |  | \| \| |  |  |  |  |  |  |  |  |
| 2 | Vitvi15g01082\_t001 |  | \| \| |  | \| \| |  |  |  |  |  |  |  |  |
| 2 | Vitvi15g01083\_t001 |  | \| \| |  | \| \| |  |  |  |  |  |  |  |  |
| 2 | Vitvi15g04609\_t001 |  | \| \| |  | \| \| |  |  |  |  |  |  |  |  |
| 2 | Vitvi15g04610\_t001 |  | \| \| |  | \| \| |  |  |  |  |  |  |  |  |
| 2 | Vitvi15g01646\_t001 |  | \| \| |  | \| \| |  |  |  |  |  |  |  |  |
| 2 | Vitvi15g01647\_t001 |  | \| \| |  | \| \| |  |  |  |  |  |  |  |  |
| 2 | Vitvi15g01084\_t001 |  | \| \| |  | \| \| |  |  |  |  |  |  |  |  |
| 2 | Vitvi15g01648\_t001 |  | \| \| |  | Vitvi02g00043\_t001 |  |  |  |  |  |  |  |  |
| 1 | Vitvi15g04611\_t001 |  | \| \| |  |  |  |  |  |  |  |  |  |  |
| 1 | Vitvi15g01085\_t001 |  | \| \| |  |  |  |  |  |  |  |  |  |  |
| 1 | Vitvi15g01086\_t001 |  | \| \| |  |  |  |  |  |  |  |  |  |  |
| 1 | Vitvi15g01087\_t001 |  | \| \| |  |  |  |  |  |  |  |  |  |  |
| 1 | Vitvi15g01088\_t001 |  | \| \| |  |  |  |  |  |  |  |  |  |  |
| 1 | Vitvi15g01089\_t001 |  | \| \| |  |  |  |  |  |  |  |  |  |  |
| 1 | Vitvi15g01649\_t001 |  | \| \| |  |  |  |  |  |  |  |  |  |  |
| 1 | Vitvi15g01090\_t001 |  | \| \| |  |  |  |  |  |  |  |  |  |  |
| 1 | Vitvi15g01091\_t001 |  | \| \| |  |  |  |  |  |  |  |  |  |  |
| 1 | Vitvi15g01092\_t001 |  | \| \| |  |  |  |  |  |  |  |  |  |  |
| 1 | Vitvi15g01093\_t001 |  | \| \| |  |  |  |  |  |  |  |  |  |  |
| 1 | Vitvi15g01094\_t001 |  | Vitvi16g04379\_t001 |  |  |  |  |  |  |  |  |  |  |
| 0 | Vitvi15g01095\_t001 |  |  |  |  |  |  |  |  |  |  |  |  |
| 0 | Vitvi15g01096\_t001 |  |  |  |  |  |  |  |  |  |  |  |  |
| 0 | Vitvi15g01097\_t001 |  |  |  |  |  |  |  |  |  |  |  |  |
| 0 | Vitvi15g01098\_t004 |  |  |  |  |  |  |  |  |  |  |  |  |
| 0 | Vitvi15g01099\_t003 |  |  |  |  |  |  |  |  |  |  |  |  |
| 0 | Vitvi15g01100\_t001 |  |  |  |  |  |  |  |  |  |  |  |  |
| 0 | Vitvi15g01101\_t002 |  |  |  |  |  |  |  |  |  |  |  |  |
| 0 | Vitvi15g04612\_t001 |  |  |  |  |  |  |  |  |  |  |  |  |
| 0 | Vitvi15g01102\_t001 |  |  |  |  |  |  |  |  |  |  |  |  |
| 0 | Vitvi15g01103\_t001 |  |  |  |  |  |  |  |  |  |  |  |  |
| 0 | Vitvi15g01104\_t001 |  |  |  |  |  |  |  |  |  |  |  |  |
| 0 | Vitvi15g01652\_t001 |  |  |  |  |  |  |  |  |  |  |  |  |
| 0 | Vitvi15g04613\_t001 |  |  |  |  |  |  |  |  |  |  |  |  |
| 1 | Vitvi15g01106\_t001 |  | Vitvi02g00303\_t001 |  |  |  |  |  |  |  |  |  |  |
| 1 | Vitvi15g01107\_t001 |  | \| \| |  |  |  |  |  |  |  |  |  |  |
| 1 | Vitvi15g01108\_t001 |  | \| \| |  |  |  |  |  |  |  |  |  |  |
| 1 | Vitvi15g01110\_t002 |  | Vitvi02g00310\_t001 |  |  |  |  |  |  |  |  |  |  |
| 1 | Vitvi15g01111\_t001 |  | \| \| |  |  |  |  |  |  |  |  |  |  |
| 1 | Vitvi15g01112\_t001 |  | \| \| |  |  |  |  |  |  |  |  |  |  |
| 2 | Vitvi15g01113\_t001 |  | \| \| |  | Vitvi16g01068\_t002 |  |  |  |  |  |  |  |  |
| 2 | Vitvi15g01114\_t001 |  | Vitvi02g00311\_t001 |  | \| \| |  |  |  |  |  |  |  |  |
| 2 | Vitvi15g01115\_t002 |  | \| \| |  | Vitvi16g01066\_t001 |  |  |  |  |  |  |  |  |
| 2 | Vitvi15g01116\_t001 |  | \| \| |  | \| \| |  |  |  |  |  |  |  |  |
| 2 | Vitvi15g01117\_t002 |  | Vitvi02g00314\_t002 |  | \| \| |  |  |  |  |  |  |  |  |
| 2 | Vitvi15g01118\_t005 |  | \| \| |  | \| \| |  |  |  |  |  |  |  |  |
| 2 | Vitvi15g04614\_t001 |  | \| \| |  | \| \| |  |  |  |  |  |  |  |  |
| 2 | Vitvi15g01119\_t001 |  | \| \| |  | \| \| |  |  |  |  |  |  |  |  |
| 2 | Vitvi15g01120\_t001 |  | \| \| |  | \| \| |  |  |  |  |  |  |  |  |
| 2 | Vitvi15g04615\_t001 |  | \| \| |  | \| \| |  |  |  |  |  |  |  |  |
| 2 | Vitvi15g01121\_t001 |  | \| \| |  | \| \| |  |  |  |  |  |  |  |  |
| 2 | Vitvi15g04616\_t001 |  | \| \| |  | \| \| |  |  |  |  |  |  |  |  |
| 2 | Vitvi15g01122\_t001 |  | \| \| |  | \| \| |  |  |  |  |  |  |  |  |
| 2 | Vitvi15g01123\_t001 |  | Vitvi02g00316\_t001 |  | Vitvi16g01058\_t001 |  |  |  |  |  |  |  |  |
| 2 | Vitvi15g01124\_t001 |  | Vitvi02g00317\_t001 |  | \| \| |  |  |  |  |  |  |  |  |
| 1 | Vitvi15g01125\_t001 |  |  |  | \| \| |  |  |  |  |  |  |  |  |
| 1 | Vitvi15g01126\_t001 |  |  |  | \| \| |  |  |  |  |  |  |  |  |
| 1 | Vitvi15g01656\_t001 |  |  |  | \| \| |  |  |  |  |  |  |  |  |
| 1 | Vitvi15g04617\_t001 |  |  |  | \| \| |  |  |  |  |  |  |  |  |
| 1 | Vitvi15g01657\_t001 |  |  |  | \| \| |  |  |  |  |  |  |  |  |
| 1 | Vitvi15g01127\_t001 |  |  |  | \| \| |  |  |  |  |  |  |  |  |
| 1 | Vitvi15g01658\_t001 |  |  |  | \| \| |  |  |  |  |  |  |  |  |
| 1 | Vitvi15g01128\_t001 |  |  |  | \| \| |  |  |  |  |  |  |  |  |
| 1 | Vitvi15g01129\_t002 |  |  |  | Vitvi16g01055\_t002 |  |  |  |  |  |  |  |  |
| 1 | Vitvi15g01131\_t002 |  |  |  | \| \| |  |  |  |  |  |  |  |  |
| 1 | Vitvi15g04618\_t001 |  |  |  | \| \| |  |  |  |  |  |  |  |  |
| 1 | Vitvi15g01134\_t001 |  |  |  | Vitvi16g01054\_t002 |  |  |  |  |  |  |  |  |
| 1 | Vitvi15g01135\_t001 |  |  |  | \| \| |  |  |  |  |  |  |  |  |
| 1 | Vitvi15g01659\_t001 |  |  |  | \| \| |  |  |  |  |  |  |  |  |
| 1 | Vitvi15g01660\_t001 |  |  |  | \| \| |  |  |  |  |  |  |  |  |
| 1 | Vitvi15g01136\_t001 |  |  |  | \| \| |  |  |  |  |  |  |  |  |
| 1 | Vitvi15g04619\_t001 |  |  |  | \| \| |  |  |  |  |  |  |  |  |
| 1 | Vitvi15g04620\_t001 |  |  |  | \| \| |  |  |  |  |  |  |  |  |
| 1 | Vitvi15g01662\_t001 |  |  |  | \| \| |  |  |  |  |  |  |  |  |
| 1 | Vitvi15g04621\_t001 |  |  |  | \| \| |  |  |  |  |  |  |  |  |
| 1 | Vitvi15g01663\_t001 |  |  |  | \| \| |  |  |  |  |  |  |  |  |
| 1 | Vitvi15g01664\_t001 |  |  |  | \| \| |  |  |  |  |  |  |  |  |
| 1 | Vitvi15g04622\_t001 |  |  |  | \| \| |  |  |  |  |  |  |  |  |
| 1 | Vitvi15g04623\_t001 |  |  |  | \| \| |  |  |  |  |  |  |  |  |
| 1 | Vitvi15g01667\_t001 |  |  |  | \| \| |  |  |  |  |  |  |  |  |
| 1 | Vitvi15g04624\_t001 |  |  |  | \| \| |  |  |  |  |  |  |  |  |
| 1 | Vitvi15g01668\_t001 |  |  |  | \| \| |  |  |  |  |  |  |  |  |
| 1 | Vitvi15g01669\_t001 |  |  |  | \| \| |  |  |  |  |  |  |  |  |
| 1 | Vitvi15g01670\_t001 |  |  |  | \| \| |  |  |  |  |  |  |  |  |
| 1 | Vitvi15g04625\_t001 |  |  |  | \| \| |  |  |  |  |  |  |  |  |
| 1 | Vitvi15g01139\_t001 |  |  |  | \| \| |  |  |  |  |  |  |  |  |
| 1 | Vitvi15g01140\_t001 |  |  |  | \| \| |  |  |  |  |  |  |  |  |
| 1 | Vitvi15g01141\_t001 |  |  |  | \| \| |  |  |  |  |  |  |  |  |
| 1 | Vitvi15g01142\_t001 |  |  |  | \| \| |  |  |  |  |  |  |  |  |
| 1 | Vitvi15g01672\_t001 |  |  |  | \| \| |  |  |  |  |  |  |  |  |
| 1 | Vitvi15g01143\_t001 |  |  |  | Vitvi16g01896\_t001 |  |  |  |  |  |  |  |  |
| 1 | Vitvi15g01144\_t001 |  | Vitvi02g00328\_t003 |  |  |  |  |  |  |  |  |  |  |
| 1 | Vitvi15g01673\_t001 |  | \| \| |  |  |  |  |  |  |  |  |  |  |
| 1 | Vitvi15g01674\_t001 |  | \| \| |  |  |  |  |  |  |  |  |  |  |
| 1 | Vitvi15g04626\_t001 |  | \| \| |  |  |  |  |  |  |  |  |  |  |
| 1 | Vitvi15g01145\_t001 |  | Vitvi02g00331\_t001 |  |  |  |  |  |  |  |  |  |  |
| 1 | Vitvi15g01675\_t001 |  | \| \| |  |  |  |  |  |  |  |  |  |  |
| 1 | Vitvi15g01676\_t001 |  | \| \| |  |  |  |  |  |  |  |  |  |  |
| 1 | Vitvi15g01677\_t001 |  | \| \| |  |  |  |  |  |  |  |  |  |  |
| 1 | Vitvi15g01678\_t001 |  | \| \| |  |  |  |  |  |  |  |  |  |  |
| 1 | Vitvi15g01679\_t001 |  | \| \| |  |  |  |  |  |  |  |  |  |  |
| 1 | Vitvi15g01680\_t001 |  | \| \| |  |  |  |  |  |  |  |  |  |  |
| 1 | Vitvi15g01681\_t001 |  | \| \| |  |  |  |  |  |  |  |  |  |  |
| 1 | Vitvi15g01146\_t001 |  | \| \| |  |  |  |  |  |  |  |  |  |  |
| 1 | Vitvi15g01147\_t001 |  | Vitvi02g01381\_t001 |  |  |  |  |  |  |  |  |  |  |
| 1 | Vitvi15g01148\_t001 |  | \| \| |  |  |  |  |  |  |  |  |  |  |
| 1 | Vitvi15g01149\_t001 |  | \| \| |  |  |  |  |  |  |  |  |  |  |
| 1 | Vitvi15g01682\_t001 |  | \| \| |  |  |  |  |  |  |  |  |  |  |
| 1 | Vitvi15g01150\_t001 |  | \| \| |  |  |  |  |  |  |  |  |  |  |
| 1 | Vitvi15g01683\_t001 |  | Vitvi02g01384\_t001 |  |  |  |  |  |  |  |  |  |  |
| 1 | Vitvi15g01151\_t001 |  | \| \| |  |  |  |  |  |  |  |  |  |  |
| 1 | Vitvi15g04627\_t001 |  | \| \| |  |  |  |  |  |  |  |  |  |  |
| 1 | Vitvi15g01156\_t001 |  | \| \| |  |  |  |  |  |  |  |  |  |  |
| 1 | Vitvi15g01684\_t001 |  | \| \| |  |  |  |  |  |  |  |  |  |  |
| 1 | Vitvi15g01685\_t001 |  | \| \| |  |  |  |  |  |  |  |  |  |  |
| 1 | Vitvi15g01157\_t001 |  | \| \| |  |  |  |  |  |  |  |  |  |  |
| 1 | Vitvi15g01158\_t001 |  | \| \| |  |  |  |  |  |  |  |  |  |  |
| 1 | Vitvi15g01159\_t001 |  | \| \| |  |  |  |  |  |  |  |  |  |  |
| 1 | Vitvi15g01160\_t001 |  | Vitvi02g00348\_t001 |  |  |  |  |  |  |  |  |  |  |
| 1 | Vitvi15g01161\_t001 |  | \| \| |  |  |  |  |  |  |  |  |  |  |
| 1 | Vitvi15g01162\_t002 |  | Vitvi02g00349\_t001 |  |  |  |  |  |  |  |  |  |  |
| 1 | Vitvi15g01686\_t001 |  | \| \| |  |  |  |  |  |  |  |  |  |  |
| 1 | Vitvi15g01163\_t001 |  | \| \| |  |  |  |  |  |  |  |  |  |  |
| 1 | Vitvi15g04628\_t001 |  | \| \| |  |  |  |  |  |  |  |  |  |  |
| 1 | Vitvi15g01165\_t001 |  | \| \| |  |  |  |  |  |  |  |  |  |  |
| 1 | Vitvi15g01166\_t001 |  | \| \| |  |  |  |  |  |  |  |  |  |  |
| 1 | Vitvi15g01687\_t001 |  | \| \| |  |  |  |  |  |  |  |  |  |  |
| 1 | Vitvi15g04629\_t001 |  | \| \| |  |  |  |  |  |  |  |  |  |  |
| 1 | Vitvi15g04630\_t001 |  | \| \| |  |  |  |  |  |  |  |  |  |  |
| 1 | Vitvi15g04631\_t001 |  | \| \| |  |  |  |  |  |  |  |  |  |  |
| 1 | Vitvi15g01690\_t001 |  | \| \| |  |  |  |  |  |  |  |  |  |  |
| 1 | Vitvi15g04632\_t001 |  | \| \| |  |  |  |  |  |  |  |  |  |  |
| 1 | Vitvi15g01691\_t001 |  | \| \| |  |  |  |  |  |  |  |  |  |  |
| 1 | Vitvi15g04633\_t001 |  | \| \| |  |  |  |  |  |  |  |  |  |  |
| 1 | Vitvi15g04634\_t001 |  | \| \| |  |  |  |  |  |  |  |  |  |  |
| 1 | Vitvi15g04635\_t001 |  | \| \| |  |  |  |  |  |  |  |  |  |  |
| 1 | Vitvi15g01693\_t001 |  | \| \| |  |  |  |  |  |  |  |  |  |  |
| 1 | Vitvi15g01695\_t001 |  | \| \| |  |  |  |  |  |  |  |  |  |  |
| 1 | Vitvi15g01168\_t001 |  | \| \| |  |  |  |  |  |  |  |  |  |  |
| 1 | Vitvi15g04636\_t001 |  | \| \| |  |  |  |  |  |  |  |  |  |  |
| 1 | Vitvi15g01169\_t001 |  | \| \| |  |  |  |  |  |  |  |  |  |  |
| 1 | Vitvi15g01170\_t001 |  | \| \| |  |  |  |  |  |  |  |  |  |  |
| 1 | Vitvi15g01171\_t001 |  | Vitvi02g00368\_t001 |  |  |  |  |  |  |  |  |  |  |
| 0 | Vitvi15g01172\_t002 |  |  |  |  |  |  |  |  |  |  |  |  |
| 0 | Vitvi15g01173\_t001 |  |  |  |  |  |  |  |  |  |  |  |  |
| 0 | Vitvi15g01174\_t001 |  |  |  |  |  |  |  |  |  |  |  |  |
| 0 | Vitvi15g01696\_t001 |  |  |  |  |  |  |  |  |  |  |  |  |
| 0 | Vitvi15g01697\_t001 |  |  |  |  |  |  |  |  |  |  |  |  |
| 0 | Vitvi15g04637\_t001 |  |  |  |  |  |  |  |  |  |  |  |  |
| 0 | Vitvi15g04638\_t001 |  |  |  |  |  |  |  |  |  |  |  |  |
| 0 | Vitvi15g04639\_t001 |  |  |  |  |  |  |  |  |  |  |  |  |
| 0 | Vitvi15g01177\_t001 |  |  |  |  |  |  |  |  |  |  |  |  |
| 0 | Vitvi15g01178\_t002 |  |  |  |  |  |  |  |  |  |  |  |  |
| 0 | Vitvi15g01179\_t001 |  |  |  |  |  |  |  |  |  |  |  |  |
| 0 | Vitvi15g01705\_t001 |  |  |  |  |  |  |  |  |  |  |  |  |
| 0 | Vitvi15g01181\_t001 |  |  |  |  |  |  |  |  |  |  |  |  |
| 0 | Vitvi15g01707\_t001 |  |  |  |  |  |  |  |  |  |  |  |  |
| 0 | Vitvi15g01182\_t004 |  |  |  |  |  |  |  |  |  |  |  |  |
| 0 | Vitvi15g04640\_t001 |  |  |  |  |  |  |  |  |  |  |  |  |
| 0 | Vitvi15g01183\_t001 |  |  |  |  |  |  |  |  |  |  |  |  |
| 0 | Vitvi15g01184\_t001 |  |  |  |  |  |  |  |  |  |  |  |  |
| 0 | Vitvi15g01187\_t001 |  |  |  |  |  |  |  |  |  |  |  |  |
| 0 | Vitvi15g01709\_t001 |  |  |  |  |  |  |  |  |  |  |  |  |
| 0 | Vitvi15g01188\_t001 |  |  |  |  |  |  |  |  |  |  |  |  |
| 0 | Vitvi15g04641\_t002 |  |  |  |  |  |  |  |  |  |  |  |  |
| 0 | Vitvi15g04642\_t001 |  |  |  |  |  |  |  |  |  |  |  |  |
| 0 | Vitvi15g01191\_t002 |  |  |  |  |  |  |  |  |  |  |  |  |
| 0 | Vitvi15g04643\_t001 |  |  |  |  |  |  |  |  |  |  |  |  |
| 0 | Vitvi15g04644\_t001 |  |  |  |  |  |  |  |  |  |  |  |  |
| 0 | Vitvi15g04645\_t001 |  |  |  |  |  |  |  |  |  |  |  |  |
| 0 | Vitvi15g04646\_t001 |  |  |  |  |  |  |  |  |  |  |  |  |
| 0 | Vitvi15g01196\_t001 |  |  |  |  |  |  |  |  |  |  |  |  |
| 0 | Vitvi15g04647\_t001 |  |  |  |  |  |  |  |  |  |  |  |  |
| 0 | Vitvi15g01200\_t001 |  |  |  |  |  |  |  |  |  |  |  |  |
| 0 | Vitvi15g04648\_t001 |  |  |  |  |  |  |  |  |  |  |  |  |
| 0 | Vitvi15g04649\_t001 |  |  |  |  |  |  |  |  |  |  |  |  |
| 0 | Vitvi15g04650\_t001 |  |  |  |  |  |  |  |  |  |  |  |  |
| 0 | Vitvi15g04651\_t001 |  |  |  |  |  |  |  |  |  |  |  |  |
| 0 | Vitvi15g04652\_t001 |  |  |  |  |  |  |  |  |  |  |  |  |
| 0 | Vitvi15g04653\_t001 |  |  |  |  |  |  |  |  |  |  |  |  |
| 0 | Vitvi15g04654\_t001 |  |  |  |  |  |  |  |  |  |  |  |  |
| 0 | Vitvi15g04655\_t001 |  |  |  |  |  |  |  |  |  |  |  |  |
| 0 | Vitvi15g04656\_t001 |  |  |  |  |  |  |  |  |  |  |  |  |
| 0 | Vitvi15g04657\_t001 |  |  |  |  |  |  |  |  |  |  |  |  |
| 0 | Vitvi15g04658\_t001 |  |  |  |  |  |  |  |  |  |  |  |  |
| 0 | Vitvi15g04659\_t001 |  |  |  |  |  |  |  |  |  |  |  |  |
| 0 | Vitvi15g04660\_t001 |  |  |  |  |  |  |  |  |  |  |  |  |
| 0 | Vitvi15g04661\_t001 |  |  |  |  |  |  |  |  |  |  |  |  |
| 0 | Vitvi15g04662\_t001 |  |  |  |  |  |  |  |  |  |  |  |  |
| 0 | Vitvi15g04663\_t001 |  |  |  |  |  |  |  |  |  |  |  |  |
| 0 | Vitvi15g04664\_t001 |  |  |  |  |  |  |  |  |  |  |  |  |
| 0 | Vitvi15g04665\_t001 |  |  |  |  |  |  |  |  |  |  |  |  |
| 0 | Vitvi15g04666\_t001 |  |  |  |  |  |  |  |  |  |  |  |  |
| 0 | Vitvi15g04667\_t001 |  |  |  |  |  |  |  |  |  |  |  |  |
| 0 | Vitvi15g04668\_t001 |  |  |  |  |  |  |  |  |  |  |  |  |
| 0 | Vitvi15g04669\_t001 |  |  |  |  |  |  |  |  |  |  |  |  |
| 0 | Vitvi15g04670\_t001 |  |  |  |  |  |  |  |  |  |  |  |  |
| 0 | Vitvi15g04671\_t001 |  |  |  |  |  |  |  |  |  |  |  |  |
| 0 | Vitvi15g04672\_t001 |  |  |  |  |  |  |  |  |  |  |  |  |
| 0 | Vitvi15g04673\_t001 |  |  |  |  |  |  |  |  |  |  |  |  |
| 0 | Vitvi15g04674\_t001 |  |  |  |  |  |  |  |  |  |  |  |  |
| 0 | Vitvi15g04675\_t001 |  |  |  |  |  |  |  |  |  |  |  |  |
| 0 | Vitvi15g04676\_t001 |  |  |  |  |  |  |  |  |  |  |  |  |
| 0 | Vitvi15g04677\_t001 |  |  |  |  |  |  |  |  |  |  |  |  |
| 0 | Vitvi15g04678\_t001 |  |  |  |  |  |  |  |  |  |  |  |  |
| 0 | Vitvi15g04679\_t001 |  |  |  |  |  |  |  |  |  |  |  |  |
